# Social Salience Discriminates Learnability of Contextual Cues in an Artificial Language

**DOI:** 10.3389/fpsyg.2017.00051

**Published:** 2017-01-30

**Authors:** Péter Rácz, Jennifer B. Hay, Janet B. Pierrehumbert

**Affiliations:** ^1^Department of Archeology and Anthropology, University of BristolBristol, UK; ^2^New Zealand Institute of Language Brain and Behaviour, University of CanterburyChristchurch, New Zealand; ^3^Department of Linguistics, University of CanterburyChristchurch, New Zealand; ^4^Oxford e-Research Centre, University of OxfordOxford, UK; ^5^Department of Linguistics, Northwestern UniversityEvanston, IL, USA

**Keywords:** salience, language variation and change, experimental linguistics, morphology, indexicality, sociolinguistics, artificial language learning

## Abstract

We investigate the learning of contextual meaning by adults in an artificial language. Contextual meaning here refers to the non-denotative contextual information that speakers attach to a linguistic construction. Through a series of short games, played online, we test how well adults can learn different contextual meanings for a word-formation pattern in an artificial language. We look at whether learning contextual meanings depends on the social salience of the context, whether our players interpret these contexts generally, and whether the learned meaning is generalized to new words. Our results show that adults are capable of learning contextual meaning if the context is socially salient, coherent, and interpretable. Once a contextual meaning is recognized, it is readily generalized to related forms and contexts.

## 1. Introduction

Studies of sociolinguistic variation show that people are able to associate linguistic patterns with a wide array of non-linguistic contexts (see e.g., Hay and Drager, 2007; Drager, 2010). What remains unclear is how these associations are learned, and whether learners discriminate these contexts in some structured manner. This learning problem is central in the sense that it sheds light on both the way contextual linguistic variation is structured and the way adults acquire it during their lifetime.

Of particular interest in this paper is the degree to which learners may attend differently to different types of non-linguistic context. Does the social salience of the non-linguistic context affect success in associative learning?

*Context* is interpreted in a number of ways in the relevant literature. For sociolinguists, the non-linguistic context is very broad. It includes the addressee and the discourse situation, as well as the speaker's attitudes and ideologies, which are conjoined to give social meaning to a given utterance (Eckert, 2008). For psycholinguists and psychologists, the context can also encompass higher-level situational attributes. In a given experiment, however, it can have a more specific interpretation, such as the visual field (Chun and Jiang, 1998) or the speaker (Kraljic et al., 2008b).

*Salience* is also interpreted in a number of ways, even within linguistics (Rácz, 2013). The core meaning is bottom-up and perceptual (a salient entity differs from its environment). We contrast this meaning of salience with *social salience*, a top-down, phenomenological concept, which encompasses the observer's background knowledge on the relevance of various aspects of the interaction (hence the term “social”). We will use the concept of salience to differentiate non-linguistic contexts that are equally complex in structural terms but are used to different degrees in anchoring linguistic variation. We use it as a general, neutral term for this distinction between contexts.

In this paper we introduce an experimental paradigm that facilitates investigation into the contextual learning of morphological patterns. Using this paradigm, we then conduct a series of six experiments that together demonstrate a very significant effect of social salience upon contextual morphological learning.

### 1.1. Denotative and social meaning

Linguistic constructions have denotative meaning and social meaning. Broadly speaking, the former is the concept that the construction denotes, while the latter is the social-cultural context of its use.

Denotative meaning does depend on the context—the denotative meanings of even common concrete nouns can vary with the topic of discussion and the use of metaphor; *bug* means something different in discussions of gardening and of computer programming. However, the social and topical dimensions of word choice are only moderately correlated (Altmann et al., 2011).

Social meaning—for example, information conveyed about who is speaking, who is being addressed, and the nature of their relationship—is more indirect and more variable than denotative meaning (Preston, 1996; Labov, 2001; Foulkes and Docherty, 2006; Silverstein, 2009). Both Labov and Silverstein note that awareness to social connotations can vary from having explicit stereotypes to no interpretation for social *indicator* variables that correlate with specific contexts in a way that is not acknowledged by the speakers.

In addition to dialect (Wells, 1982) and social group (Eckert, 2000), robust factors that influence social variation in language include age, gender, and sexual orientation (Labov, 2001; Pierrehumbert et al., 2004; Tagliamonte and Roeder, 2009). An important aspect of the social context is the addressee, or the speaker's relationship with the addressee (within its context). Linguistic accommodation to the addressee is a well-researched phenomenon (Coupland et al., 1991; Soliz and Giles, 2014). Certain languages, like Djirbal, develop lexical sets that are used with addressees belonging in specific kinship groups (Dixon, 1980).

Social meaning also varies in even more nuanced ways, as speakers dynamically use social-contextual information to take stances and negotiate in social interactions—different linguistic choices reflect the individual's linguistic experience and construction of social identity (Milroy, 1980; Eckert, 2000).

Listeners can, in turn, use such patterns to infer speaker characteristics or to adapt to different speakers when processing speech (for review of relevant literature see Hay and Drager, 2007; Foulkes and Hay, 2015). Some words are statistically associated with older speakers and others with younger speakers; sensitivity to these associations can be displayed through varied ease of psycholinguistic processing without any explicit awareness of age-based patterns on the individuals' part (Walker and Hay, 2011). Listeners are able to associate different speaker personae with different combinations of morphological and phonological variables, based on fine-grained patterns in the ambient language (Campbell-Kibler, 2011). Individuals can also shift their categories of speech sounds based on cues of the broad cultural context, even if these cues are only peripherally present (Hay and Drager, 2010).

These examples show that social meaning can be attached to linguistic constructions that are not specific words or phrases—it can be generalized across linguistic patterns. It can apply to contexts of various levels of abstractness and expand to new contexts. It encompasses a wide variety of linguistic detail, from phonetics to word choice (Säily and Suomela, 2009; German et al., 2013). It relies on some contextual differences more heavily than on others, and speakers use it in a complex and subtle way to express intent and create a public persona (Eckert, 2012).

While numerous sociolinguistic and anthropological studies have revealed the importance of social meanings in language, less is known about how they are learned. Our understanding of social cognition in language leaves many unanswered questions about what details are noticed and remembered (Silverstein, 2009), as well as about what factors support generalization to new forms or new situations (Pierrehumbert, 2006). Foulkes (2010, p. 6) laments the lack of understanding of learning and storage of social meaning, stating that: “it now seems uncontroversial to conclude that social information is retained in memory alongside linguistic knowledge. Questions remain, however, over what sorts of social information are learned and stored, where and how they are stored in relation to linguistic information, and how social information affects linguistic processing.”

### 1.2. The role of the context in learning linguistic categories

If context is important, how do we learn to use it?

The contextual learning literature that is most relevant to this paper focusses particularly on the role of the broader extra-linguistic context—above and beyond the referent—in learning a linguistic category. Three important findings emerge from it.

First, consistency across contexts aids recall: a category is remembered more accurately in the context in which it was originally learned. In word-learning tasks, words are retrieved more accurately if recall occurs in the same location as training. Godden and Baddeley (1975), for example, show that words learned underwater are more accurately retrieved underwater. This relates to more general work on memory retrieval, where it has been shown many times that consistency of contextual information between encoding and retrieval leads to increased recall. Smith and Vela (2001) review literature showing that “people tend to be aware of their surroundings even when memorizing something. As such, environmental features are typically encoded along with the to-be-remembered material.” Recurrent information will also invoke the context it was acquired in. Models of category learning and memory retrieval (Ratcliff, 1978; Grossberg, 1987) operate using notions of context-specificity. Certain memories activate specific contexts in which they were learned.

Broad experimental work in psychology discusses the role of contextual cues in category learning (Chun and Jiang, 1998; Goujon et al., 2015). This work shows that visual decision tasks speed up if the trial (with a given visual context) is shown repeatedly, despite the fact that participants are unable to identify the contexts afterwards, suggesting that the effect of the context on learning can be implicit. Qian et al. (2014) show that in a “whack-a-mole” type game, players are faster at predicting the location of the mole if the location is probabilistically cued by moving background images that the player is not overtly oriented to. Gómez (2002) note that a consistent structure is learned better across multiple contexts. Observing the same pattern across multiple speakers improves learning as well (Rost and McMurray, 2009). Individuals use contextual memory to aid recall and prediction. Lleras and Von Mühlenen (2004) revisit Chun's paradigm, and their results indicate that the success of contextual cues depends on whether participants are focussing on the task in a narrow sense (presumably discarding the context) or are trying to take a holistic approach.

Second, categories learned in one context can generalize to another, similar context. Van der Zande et al. (2014) show that phonetic categories that shift due to exposure to a speaker retain this shift even when listening to another speaker. Maye et al. (2008) extend this generalization to a new accent: their participants are able to use a context-specific vowel adaptation mechanism to process phonetic variation coming from a speaker and then, in turn, re-use the adapted vowel categories when encountering a similar speaker. Kraljic and Samuel (2006) show that a phonetic category distinction is generalized to a new linguistic context and also to a new speaker. That is, even if participants are trained on the distinction in one speaker voice, they carry over the distinction to another voice.

Third, listeners do not treat all available information the same way. Kraljic et al. (2008b) find that, while we first learn all phonetic detail as characteristic of a given speaker, we are later able to re-assess this knowledge and discard contextual variation that is based on an arbitrary idiosyncrasy of the speaker (such as talking with a pen in the mouth). Kraljic et al. (2008a) show that phonetic variation is processed differently if it is due to a consistent idiosyncrasy of a speaker (like a speech impediment) than if it represents a dialectal contextual allophone. Leung and Williams (2012) show that a distinction based on the animacy of referents is learned much more easily and generalized more readily than a distinction based on size differences between referents. Similar results have been found even for purely phonological contexts. Becker et al. (2011) find that Turkish speakers apply some statistical regularities in the Turkish lexicon, but not others, in a forced-choice wugs task; they conclude that speakers distinguish accidental from well-grounded statistical generalizations.

We are able to associate linguistic categories with non-linguistic contexts, even if these contexts are fairly arbitrary. We can extend this knowledge to similar but different contexts as well. And yet, we do not rely on all differences the same way—we distinguish information that is *relevant* in the context from information that is *accidental* to it.

### 1.3. Weighing the social salience of contexts

The amount of detail observed in sociolinguistic variation (Hay and Drager, 2007), coupled with memory models (Nosofsky, 1988), suggests that language users are able to construct social meaning based on a vast number of contexts. Some assume, however, that human memory is too restricted for this. Therefore, at least some of the information may be discarded if it cannot be used to make generalizations and if it taxes resources overtly (for the debate, cf. Gluck and Myers, 2001; Denton et al., 2008; Baayen et al., 2013).

How do we choose between useful and irrelevant contextual information? While the statistical co-occurrence of contexts and patterns is important, this is not the complete story. Selective attention guides which details are more important in processing information (Itti et al., 1998). Variation can be interpreted differently depending on its source (Kraljic et al., 2008a).

Relevance in turn derives from complicated assumptions about how the world works: that some speaker differences are consistent and others are haphazard, and that some contexts are more informative of language variation than others. When these include assumptions about what sorts of people are members of the same group, they rest on social constructs or categories. What information is grouped together and what is discarded both play a role in structuring social-contextual language variation.

Many experimental studies have explored the associations between familiar social groups and accentual features. Relatively few studies have investigated learning that involves novel social groups, or learning of socio-linguistic cues other than ones at the phonetic level. This may be because it is a daunting task to set up scenarios in which the relationship between the social context and the linguistic pattern is transparent and well-controlled. However, there are several noteworthy studies. Work by Docherty et al. (2013) and Langstrof (2014) find that people can associate familiar dialectal variables with arbitrary “tribes” in a laboratory setting. Roberts (2008) shows that people are able to come up with morphological markers in a nonce language in order to demarcate in-group and out-group membership in a laboratory setting. Beckner et al. (2016) find that participants shift their linguistic patterns to accommodate to a group of human peers but not to a group of humanoid robots. The Beckner study shows extension of the accommodation pattern to new words that are similar in form to previously encountered ones. These studies do not look at extension or generalization to different speakers.

The term *relevance* often implies conscious decision making on which contexts to consider and which to discard. Work on phonetic learning, however, suggests that we discriminate contexts largely implicitly. We will use the term *social salience* to compare the “usefulness” of a context for linguistic learning and—as a consequence—people's ability to rely on it.

### 1.4. Individual variation in contextual learning

As in any learning task, people's success rates will vary in contextual learning. Work on language variation and change provides an important body of evidence on how people learn linguistic patterns that are associated with non-linguistic contexts. Labov (2001) shows that a new sociolinguistic variant does not diffuse uniformly through the population. Typically, there are community leaders of language change, who are chiefly responsible for spreading an innovative variant in the community. Later work distinguishes components of this process, all of which are relevant here. First, computational models have led to the conclusion that an innovative variant succeeds only if it carries a positive social weight, something which of course depends on learning a social association for the variant in the first place. (cf. e.g., Baxter et al., 2009; Fagyal et al., 2010). However, this positive weight does not need to be present in the minds of everyone in the community, but only in the minds of a critical group of early adopters—people who take up and use the new variant before other people do (Pierrehumbert et al., 2014). Experimental evidence for the existence of linguistic early adopters is found in Schumacher et al. (2014), an artificial language learning experiment in which some participants adopted an unexpected number-marking system far more than others.

These results indicate that individual variation in contextual learning is far from being a footnote example of differences in task completion—it is significant for patterns of language variation and change.

The source of such individual differences, then, becomes crucial, but it is relatively unclear. Some individuals are likely better at recognizing or remembering contextual patterns than others. For verbal and non-verbal statistical learning tasks, Siegelman and Frost (2015) show that individual performances in a verbal or a non-verbal task are not strongly correlated with different measures of intelligence and cognitive capacity. In fact, Siegelman and Frost find little correlation across performance in different statistical learning tasks. The analysis of Lleras and Von Mühlenen (2004) of individual participant behavior in their learning experiments (adopted from the work of Chun and Jiang, 1998) indicates that participant success in a task depends on the strategy adopted by the participant. This high-level, complex decision is unlikely to be derived from any single cognitive or linguistic measure. However, some systematic effects have been identified. Vocabulary size is a good predictor of how easily new word forms are learned in children (Henderson et al., 2015 and therein). Henderson et al. (2015) do not find an effect in adults, but in a related pseudo-word rating study with more statistical power, Needle et al. (2015) do find that high-vocabulary adults have a better ability to decompose nonce compounds such as *angstroof*. Brooks et al. (2016) shows that learning and generalizing an L2 morphological pattern can be partially predicted by measures of non-verbal intelligence and statistical learning ability.

Ramscar et al. (2014) argue that the life-long accumulation of experience affects performance in psycholinguistic tasks. Older people have more prior experience, and so work with a denser cue space in verbal tasks. In an explicit learning task with feedback, Metcalfe et al. (2015) find that older participants perform better, especially on unfamiliar items. Event-related potentials for the older participants indicate better ability to focus attention on feedback.

In short, participant accuracy is highly variable in learning tasks and this variation derives from a complex set of cognitive differences. However, two studies (Ramscar et al., 2014; Metcalfe et al., 2015) point to age as an interesting factor. Prior experience may affect performance on psycholinguistic tasks, either via richer mental representations gained through experience, or though better proficiency in allocating attention. Recruiting participants on Amazon Mechanical Turk provided us with a participant pool of diverse ages that makes it possible to assess this factor.

Diverse sources of evidence indicate that language use relies on contextual cues, and that speakers evaluate these cues both implicitly (based on salience and statistical co-occurrence) and more explicitly (based on social salience). How this behavior is learned is less clear, but the learning process and the individual differences manifested in it are both very relevant to the study of language variation and change.

## 2. Aims

In this paper, we report on a series of experiments that build on previous results in context-specific category learning. In the experiments, participants have to learn linguistic patterns that depend on the context. The context can be linguistic—the choice of a suffix depends on the shape of the stem. It can be non-linguistic—the choice of a suffix depends on the conversation partner. Conversation partners can differ across various dimensions. Both the patterns and the contextual differences are more abstract than those explored in studies of phonetic adaptation such as Kraljic and Samuel (2006).

The linguistic patterns we look at are morpho-phonological. They are suffixation patterns in a simple artificial language that mark the diminutive or the plural. These are both transparent, iconic relations that also show considerable variation in English and other languages. The specific linguistic contextual pattern we use (the suffix vowel should match the stem vowel) is not found in English, so participants must learn it. Their success in doing so provides the baseline condition for the experiment, serving to validate the paradigm and shed light on the strengths of the effects found for the various social factors.

The social dimension we focus on is socially robustly interpretable, the gender of the conversation partner. We contrast it with a dimension that has similar visual prominence but lacks its social salience, the spatial orientation of the conversation partner. We chose to explore the gender distinction because it is a very robust sociolinguistic marker. Children as young as 6 months, for example, preferentially match sex-cued voices and faces (Walker-Andrews et al., 1991). Sex and gender have a complex effect on the use of social meaning in general (Milroy and Milroy, 1993; Cheshire, 2002). Our experiments build on each other to provide a solid foundation for the salience of this distinction, by showing that it holds up across differing amounts of exposure, types of extensions, or types of linguistic patterns.

We ask the following questions:

1. Given a morpho-phonological pattern, how quickly and well are participants able to learn its association with a linguistic context? Are they able to generalize the pattern to new words? How does generalization to new words compare to words seen in training?
2. How well can participants associate a morpho-phonological pattern with a social context: conversation partner gender? Are they able to generalize the pattern both to new words and to new instances of the appropriate context?
3. Are all types of social association equally learnable and generalizable? Or is an incidental social property (spatial orientation) processed differently from a more static conversation partner characteristic (gender)?
4. How do individuals vary in learning contextual associations for linguistic patterns?
5. Are older participants more successful, or less successful, at learning such social contextual associations?

As we will see, for the morpho-phonological patterns we look at, learning is possible for both linguistic and non-linguistic contexts. For non-linguistic contexts, participants are more successful in learning an association with a robust, salient context, conversation partner gender. This context is interpreted broadly—gender is recognized as the defining dimension. The morphological pattern is recognized and extended to previously unseen words after training. Older participants are better learners in our data.

We find these results with more types of conversation partners (such as children and adults) and with two distinct morphological patterns, the diminutive and the plural. These results indicate that the salience of conversation partner gender *vis-à-vis* spatial orientation is a broad and general phenomenon.

## 3. Overview of experiments

Our experiments use a training-test paradigm based on a simplified version of adaptive tracking. The adaptive tracking paradigm described in Leek (2001). It was previously adapted to linguistic research by Schumacher et al. (2014). We discuss the paradigm in detail in the Methods Section of Experiment I.

Experiment I trains participants on a morphological pattern, presented visually. They see picture pairings, with a large and a small version of the same entity. The large version is named. They have to choose the name of the small version, which is a suffixed form of the name of the large version. There are two suffixes, and the correct one includes a vowel that matches the stem vowel. We find that participants learn to consider this context easily and also extend the pattern to new items.

In Experiment II the same morphological pattern is presented, again with different conversation partners. This time the pattern depends on the conversation partner. There are two conversation partners—a male and a female—and both are presented in two ways visually, in side view and in front view. One group of participants is trained with the morphological pattern depending on conversation partner identity, cued by gender (answers for conversation partner A or B pattern together) The other group is trained with the pattern depending on conversation partner spatial orientation (answers pattern together according to whether the conversation partner is presented in front view or side view).

We find that an association of the pattern with the identity of the conversation partner is easier to learn than an association of the pattern with the spatial orientation of the conversational partner. This result resembles the findings by Kraljic et al. (2008b) on learning of incidental vs. characteristic patterns of phonetic variation. The morphological pattern is interpreted broadly—in the test session, it is extended to items not seen in the training session.

Experiment III expands the scope of contextual learning to examine whether participants generalize on the basis of conversation partner gender. Gender is one of the most widely discussed predictors of sociolinguistic variation. Morpho-phonological and lexical variation that depends on gender is not restricted to languages like Dyirbal. Languages like French and German use different adjective conjugations depending on the referent, including first and second person referents in the discourse, while stochastic differences for gendered language use have been found in English as well (Hay and Walker, 2013). We find that gender is a better cue than spatial orientation. Naming is readily extended to new conversation partners that fit this context (i.e., as another female or male conversation partner).

Experiment IV focuses on the way participants rely on the denotative and the contextual aspect of the naming pattern. The general layout is similar to experiments I–III. However, the test phase is different. Instead of a right and a wrong answer, they are forced to choose between one answer that is correct in its denotative aspect but wrong in its contextual aspect and another one that is set up the other way around. We find that if the contextual cue is conversation partner *gender*, participants have split preferences between the denotation and contextual cue. With a spatial cue, they overwhelmingly prefer the denotative aspect.

In experiments V and VI we extend the paradigm to investigate a new morphological pattern—the plural—and investigate the effect of a radically increased training set size. We find that learning the plural is similar to learning the diminutive, though the results are not conclusive. Increased training improves participant accuracy in test.

## 4. Experiment I

Experiment I establishes our experimental paradigm and investigates the role of the linguistic context in learning within this paradigm.

In Experiment I, participants learn a morphological pattern that is sensitive to the linguistic context. It is a vowel harmony or partial reduplication pattern (common in the world's languages, though not present in English): the vowel of the suffix has to match the vowel of the suffixed stem. The version of adaptive tracking presented here was used successfully by Schumacher et al. (2014), who also recruited participants on Amazon Mechanical Turk. Our design, however, differs in its overall theme, as well as in the amount of training participants receive.

We explain our design in detail in Section 4.2 and address changes to it in subsequent Methods sections.

### 4.1. Participants

The experiment was hosted on Amazon Mechanical Turk (AMT). 47 people participated in the experiment. 22 are women, 25 men. All are native speakers of American English. We base this claim on the fact that all participants had IP addresses from the United States and self-identified as native speakers (those who did not were excluded from the results). The mean age is 31 years, with a standard deviation of 8.5. Participants were paid three dollars upon completion of the task.

For each of our experiments, we used Amazon Mechanical Turk worker IDs to exclude participants who had taken part in any of the other experiments. US worker IDs are independently verified by Amazon, making it very difficult for the same person to operate multiple accounts.

As in all following experiments, we used training speed to remove outliers (cf. below). For each across-subject condition, we removed the 2.5% of participants who took the most trials to finish training—i.e., the slowest ones. We filtered participants within the across-subject conditions, since we expect conditions to vary in length. In Experiment I, which has one across-subject condition, we removed 2 participants and report data from 45 participants. We return to the training phase and discuss our exclusion criteria in detail in the Results section of Experiment I.

For each experiment, we do not report the precise ratio of AMT workers who picked up the task vs. workers who finished it, since an online task can be interrupted for various reasons, including connection issues, disruptions, etc. On the whole, about 5% of the workers who started these experiments did not complete them. This, in our experience, is not an excessively high number for an online experiment.

By using Amazon Mechanical Turk, a burgeoning forum for psycholinguistic research (Munro et al., 2010), we were able to recruit a large number of participants in a short span of time. Amazon Mechanical Turk is especially fit for our experiment, which has a “game-ified,” button-input design. The game format allows for immersion of the participants, and increases the likelihood that they pay attention to the task—otherwise they cannot finish it. *Gamification* has been increasing in popularity in data collection in recent years (Von Ahn, 2006) and it has been used successfully in linguistic experiments as well (Fedzechkina et al., 2012; Schumacher et al., 2014). Relying on Amazon Mechanical Turk allowed us to run substantial numbers of subjects, so as to be able to see important differences across conditions in how likely our various predictions are borne out. Crump et al. (2013) show that more complex laboratory tasks on category learning can be replicated using subjects on AMT. However, AMT subjects are, overall, less successful learners than laboratory participants, possibly because they are less focused and attentive when participating from their homes, without the presence of an experimenter.

Experiment I tests for a main effect of a single across-subject condition while Experiment II has two across-subject conditions and tests for interactions as well. This is why the latter has twice as many participants as the former. The same logic was applied to subsequent experiments. This is an economical use of participants, but does have the restriction that power to estimate participant-level effects (here, gender and age) will vary across experiments. We return to this issue in Section 8, in which we estimate these effects on a merged dataset.

This and the following experiments reported in the paper have been overviewed and approved by the Institutional Review Board of Northwestern University and the Human Ethics Committee of the University of Canterbury. During the time of data collection, the experimenters were not affiliated with any other institutions.

### 4.2. Methods

**Figure 1:**
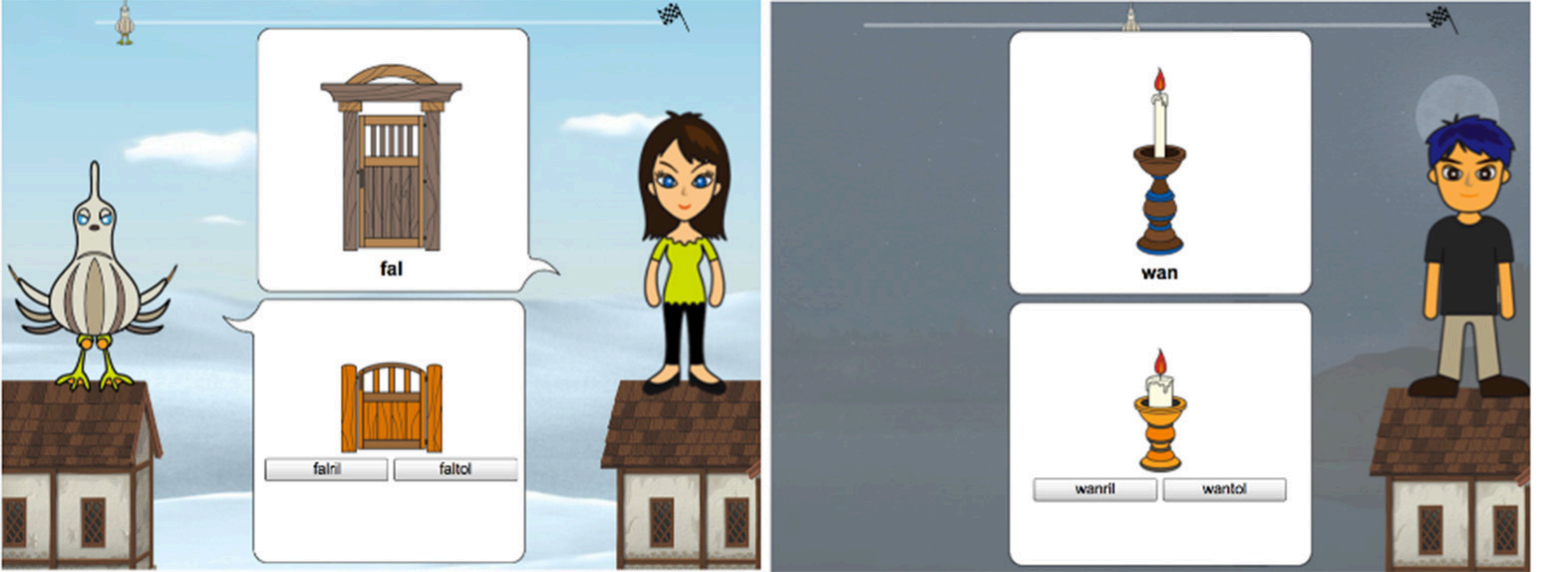
**(Left)** The in-game set-up of the training phase in all our experiments. The player is on the left, the conversation partner is on the right. The query is in the speech bubble that belongs to the conversation partner. The response choice buttons are in the speech bubble that belongs to the player. One of the answers is correct, the other one is wrong. This is a general example of the layout. In Experiment I, the correct answer depends on the stem vowel of the prompt. In the rest of the experiments, it depends on the conversation partner (as in this example). **(Right)** The test phase. The visuals separate it from training.

In Experiment I, participants play a computer game in which they have to help a bird flying roof to roof to return to its nest. The game consists of a training phase, followed by a test phase. The targets are presented in the following way. For a given *target*, the participant sees a *conversation partner* who shows a *query picture* to the main character, the bird, along with the *prompt*, the *name* of the depicted object. The bird responds with a *response picture* and *two possible names*. The participant has to choose one of them. The response picture is always the *diminutive* version of the conversation partner's picture (depicted as a small or juvenile version of the conversation partner's picture). This implies that one of the two possible names is the correct name of the diminutive of the query picture. A *target* is the combination of an item (a query-response picture pair) with a conversation partner. Figure 1 shows the general layout with examples of the phases and the mechanics. Stimuli are *visual* only.

During training, targets are presented in a random order to each participant, and the participant has to give a correct answer for every target in order to move to the next target. If they give an incorrect response, they have to return to the previous target. The test phase does not use adaptive tracking. Here, targets are presented, again, in random order. The targets in the test phase include both the training items and previously unseen items. No feedback is given. Training takes place during the in-game day, test during the in-game night. Participants are also told when they enter the test phase. The way training relies on simplified adaptive tracking guarantees that each participant has responded to each stimulus correctly at least once before moving to the test phase. Unlike training protocols with a fixed number of trials, it provides an opportunity for participants who find the task difficult to improve by training for longer.

The name of the query picture is a nonce word with a CVC structure. These are drawn from a set of 12 syllables (cf. Table 1). Half of the name syllables have the <e> vowel, the other half the <a> vowel. The two possible names of the response picture are the name of the query picture plus one of two suffixes—*pek* and *pak*. These suffix syllables were selected such that one had <e> and one had <a>. The correct response always matches the vowel of the prompt. This echoes vowel harmony or partial reduplication systems commonly found in natural languages. Participants encounter six items in training and these six items plus six new items in test. These are items that do not occur in training.

**Table 1:** **Stimuli set, Experiment I**.

fek	ran	wek	fal
pel	ral	tek	rak
tas	fan	wen	fes

We designed the stimuli with the following principles in mind: (i) the syllables should be distinctive; (ii) they should consist of a small set of frequent letters; (iii) they should be easy to pronounce for our participants, who are American English speakers; (iv) the consonant clusters in the two-syllable words should cue English word boundaries in a uniform manner. These are somewhat competing requirements but our aim was to provide a relatively optimal set that balances all of these considerations.

The names of the individual objects are randomly assigned for each participant, using the set of twelve syllables in Table 1. Six occur in training and then also in the test. Six occur only in test.

In all our experiments, participants come across four conversation partner images during the game.

**Figure 2:**
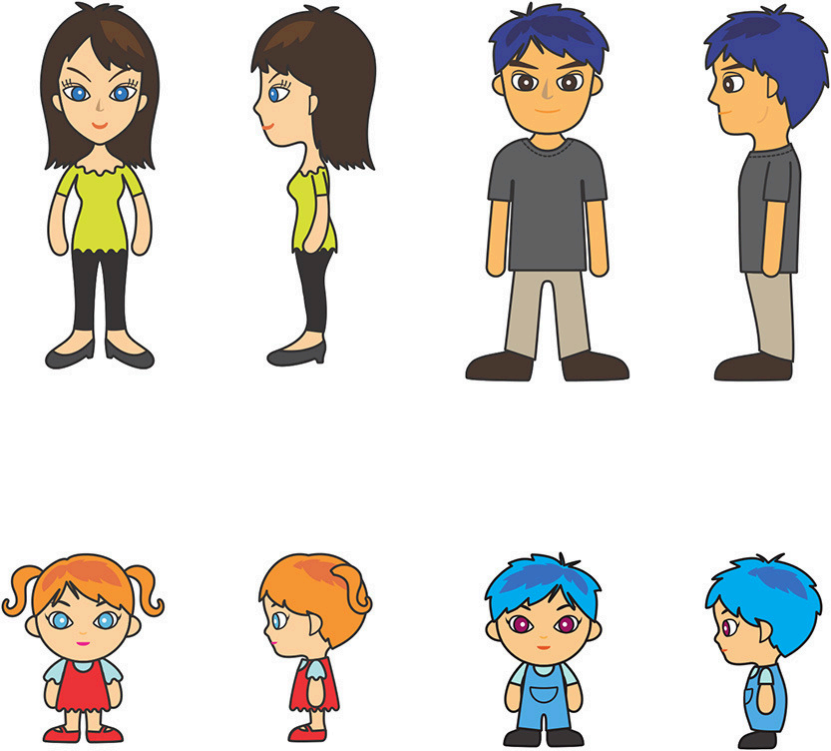
**Distances between the eight conversation partner images**.

The images we use for our conversation partners in experiments I–VI can be seen in Figure 2. We will refer to them in the paper using the labels *woman, man, girl*, and *boy*. Each figure has two perspectives, *front* and *side*, giving us 8 conversation partner images in total.

The particular images we used were designed to be matching in many respects, while still appearing visibly different according to gender and/or view. It is difficult to assess the degree to which we were successful with this aim, as human raters completing an explicit similarity rating task would be unable to avoid bringing their social knowledge to bear. However, in order to attempt an objective test that there were not strong visual differences between the different dimensions, we computed the Levenshtein distance between uniformly binned histograms of the grayscale versions of the images using Matlab (Mathworks, 2016). Histogram comparison is a common method in image processing (Pele and Werman, 2010).

**Figure 3:**
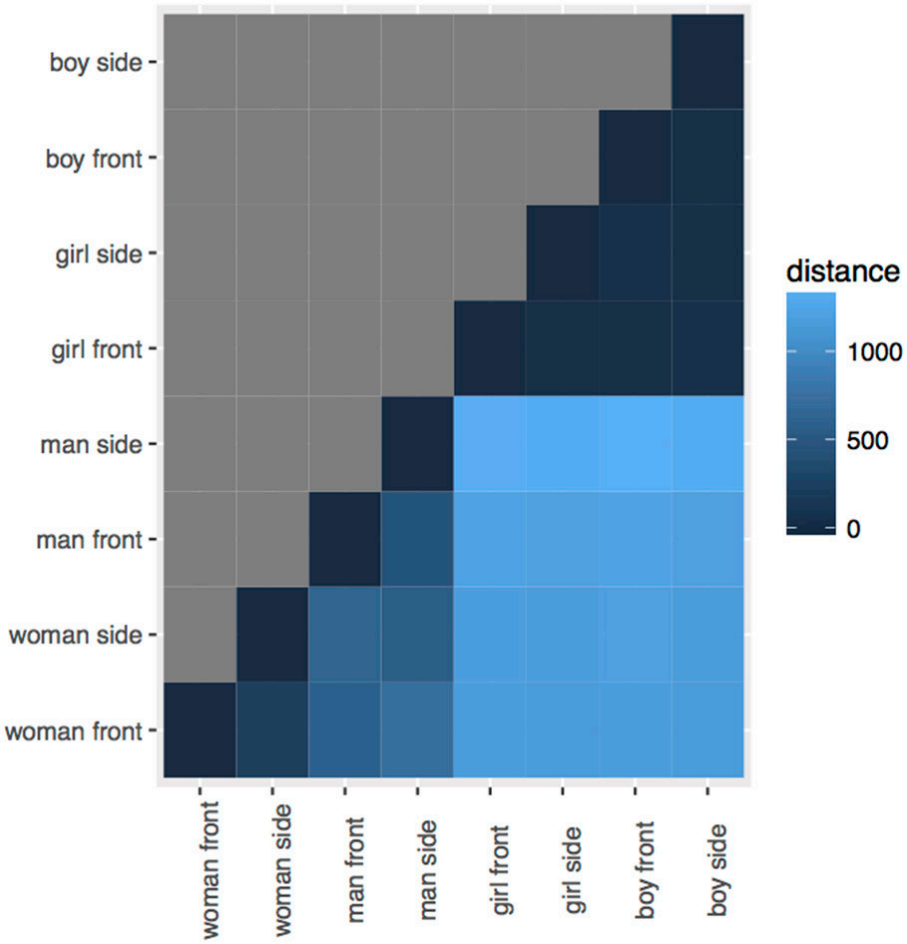
**The eight conversation partner images used in the experiments**.

The Levenshtein distance between the images is roughly similar. For the adult images, there is a slightly larger distance between the woman and the man than between the front and side views for either the woman or the man. But for the child images, the order of the distances is interwoven between the grouping factors, and the largest difference is between the boy front and side view, and the smallest difference is between the girl and the boy front views. (Figure 3 is a tile plot that shows image distances. Darker hue means smaller distance).

This visual similarity metric patterns differently for the adult and child figures, but—as we will report in the following sections—none of our experiments reveal any difference in whether participants were trained on the adult or the child images (c.f. experiments III; V). It therefore seems very unlikely that patterns relating to image similarity are driving the behaviors that we observe.

The four conversation partner images in Experiment I are the *woman* and *man* figures in Figure 2, viewed from the *front* and the *side*. All items occurred with all conversation partners, giving a minimum of 24 trials in training and 48 trials in test. Who the conversation partner is has no bearing on the correct name selection in training, since the latter only depends on vowel quality. Linguistic context is relevant, non-linguistic context is irrelevant. The conversation partners will become relevant in Experiment II. The “comic book” setup of the experiment allowed us to freely combine text with conversation partner images in all experiments.

In our experiments, the visual field of the experiment (the window in which it takes place on the user's computer screen) is the non-linguistic context. The words that occur in this visual field (written in the latin alphabet) constitute the linguistic context. We use the visual display to manipulate a classic sociolinguistic factor, the *addressee*.

### 4.3. Participant instructions

The experiments are designed to create a setting for linguistic or socio-linguistic learning that is controlled, yet still somewhat naturalistic. The task itself is made explicit, but the potential cues for the correct answers are not. Participants need to work out which cues to attend to from the potential cues available. These include the orthographic shapes of the word forms, the item pictures, and the conversation partner pictures (since the protagonist and the background are held constant). This makes the task harder. But it also makes it analogous to problems we encounter in language use, an issue that has received considerable attention in the literature of contextual language learning (cf.Yu and Smith, 2007).

Participants receive written instructions at the beginning of the game. They are told that the bird is the protagonist (“our hero”), and that they need to help our hero return to its nest by flying from roof to roof. The hero will meet people who stand on the roofs and ask questions. The hero needs to answer the questions correctly in order to proceed. The questions are explained: the person names an object and shows our hero a smaller version of the object as well. Our hero has to guess what they would call the small object. It is explained that a second phase follows this first phase. In this phase, participants need to guess the names given to small objects, just like they did in the first part. They are asked to try to remember what the right answers were in the first part, and guess the right answer based on that.

### 4.4. Hypotheses

We hypothesized that participants would learn the association of the morpho-phonological pattern with the linguistic context and generalize it to new items. Based on related studies of phonetic learning, such as the study of an indexical allophonic pattern reported in German et al. (2013), we also predicted higher accuracy for test items seen in training than for unseen test items. We also evaluated age and gender as potential predictors of performance.

### 4.5. Results

Overall, the results show that many participants succeeded in learning and generalizing. This outcome is reflected in the time course of training and in accuracy in the test phase.

Since the length of training depends on the participant's success at the learning task, training length is a good indicator of task difficulty. It is also a good indicator of participant attention and ability.

We use trial counts to express training duration. While individual trials vary in duration (there is no time limit on trial length, that is, people can spend as much time as they want on their decision), they do so to a modest degree (in Experiment I, mean (*m*) = 16 seconds, standard deviation (*sd*) = 12 seconds).

We prefer trial count to duration in time because the latter can be affected by user computer problems, server lag, and participant behavior (taking a break, answering the phone, etc.).

In similar experiments the accepted norm is removing participants who are 2 or 2.5 standard deviations outside the overall mean. This method has its problems, as shown by Leys et al. (2013), who recommend mean absolute deviation instead. For our data, neither the standard deviation threshold, nor the mean absolute deviation threshold are applicable. We decided to use a percentage threshold since trial length in our experiments is not normally distributed, making standard deviation a poor measure of the distribution of participant trial count. The distribution starts at 24 (the minimum possible number of trials) and has a long right tail. A method of outlier removal that relies on the 2 standard deviations threshold would remove about 5% of the participants from the experiment. Our method removes the slowest 2.5%. A participant cannot finish too quickly, and so the distribution of training trial counts has no left tail. We remove outliers to safeguard against participants with very poor attention.

In all experiments, we filtered participants within the across-subject conditions because we expected these to vary in length. We used a quantile threshold to remove participants in the right tail of the training trial count distribution. For every condition, we establish the 0.975 quantile threshold of the distribution of training trial counts. We exclude participants over this threshold. The number of participants removed for each experimental condition ranges between 1 and 2. Outliers for the separate conditions add up to the sum of outliers for each experiment.

In Experiment I, 2 out of 47 participants are over the 97.5% threshold.

Participants finish training much faster than a player would by chance (*m* = 43, *sd* = 18). Individual variation for trial counts is large. Participants recruited through Amazon Mechanical Turk vary more in their behavior than would the college students recruited for a typical lab experiment.

Experiment I has one across-subject condition. Training speed in this condition is only informative inasmuch as it is, on average, much shorter than what we expect if participants were guessing. This shows that some form of learning is taking place in training^1^.

**Figure 4:**
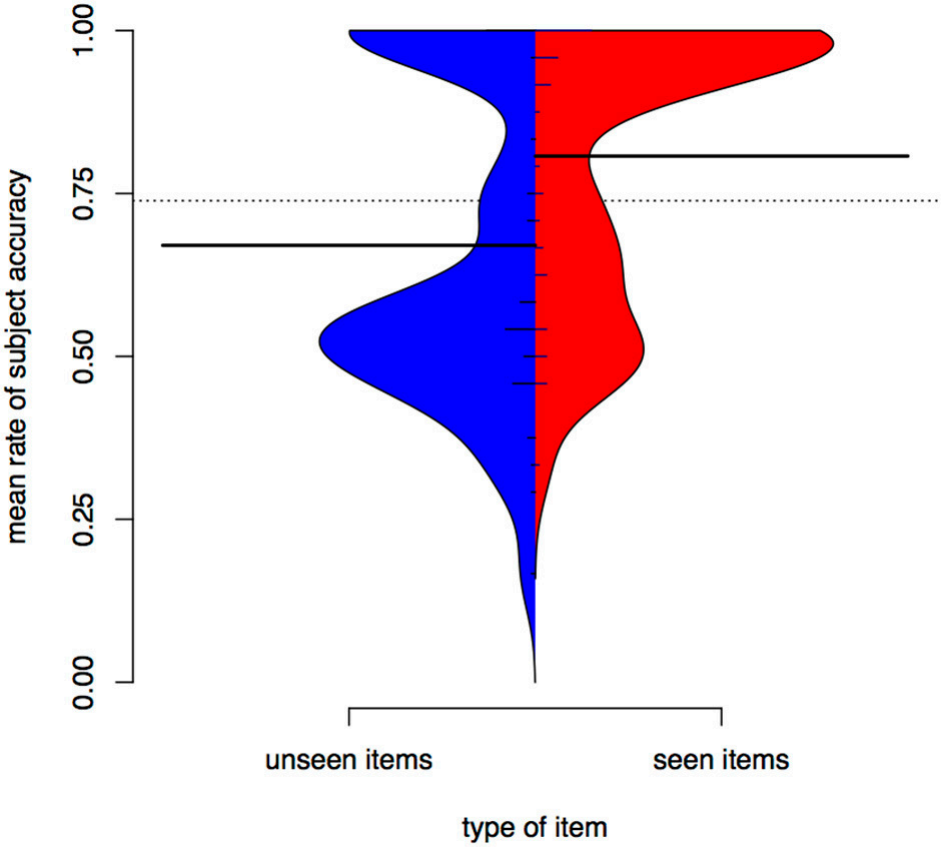
**Distributions of participant responses on previously seen and unseen test items, Experiment I**. Black horizontal bars show the mean accuracy for each set of items. The dotted line shows the overall mean. Small horizontal lines show individual values; longer if multiple individuals have the same average.

Accuracy in test depends on whether the item was seen in training. Figure 4 is a bean plot of participant accuracy in the test phase, grouped by whether the item was seen in training.

The bean plot shows the distributions of participant responses along the y axis. Mean accuracy is higher for items seen in training (seen items, right) than for items participants only encountered in test (unseen items, left). This is indicated by the long black horizontal bars. The *distribution* of mean subject accuracy rates, however, is also revealing. For items not seen in training, we see a very clear bimodal distribution, with most subject means centred either around .5 or near 1 (up to 1, actually, since it is impossible to have a *higher* accuracy rate than 1). A person whose accuracy is around 0.5 in a task that involves binary choices is effectively guessing. A person whose accuracy is 1 has done a perfect job. For items seen in training, there also appears to be a bimodal distribution, but the total mass of the upper mode is greater and more participants perform at accuracies around 0.6 to 0.7.

We used the R statistical computing environment for our analyses (R Core Team, 2016). We created our plots using ggplot (Wickham, 2009).

We stepwise fit a binomial mixed-effects regression model on the test data, using response to individual items (*correct* or *not correct*) as an outcome variable and *presence in training* and participant *age* and *gender* as predictors, with a participant grouping factor (random intercept) (Gelman and Hill, 2006; Bates et al., 2012). We used a random intercept for participants to account for participant-specific differences in variation. Since object-name pairings are generated on the fly, these are different for each participant. As a consequence, we did not need to model item-level variation (e.g., with an item random intercept), making our models computationally more effective.

For each regression model in this paper, we started with a fully specified model including all interactions and removed non-significant predictors one by one, testing for model fit using analysis of variance and the Akaike information criterion (AIC). Where a combined model was too complex we fit interactions of participant-level predictors (age and gender) and experimental conditions (cue type, item presence in training, etc.) separately. We only report the best model, which means that we exclude predictors that were not significant.

Eight out of 498 participants (across the 6 experiments) did not disclose their age in the pre-test survey. When we tested age as a predictor, we re-fit models excluding these missing data and performed analysis of variance checks on these models to inform model selection. Models excluding participants with missing data were consistent with models fit on full data. For models for which age was justified as a predictor, the reported models exclude the few participants for which we have no age data. This model selection process assures that (i) we use all the available information in our models and that (ii) participant-level and experiment-level factors, along with their interactions, are tested in each experiment.

The best model for the test phase of Experiment I can be seen in Table 2. The model includes participant age. For Experiment I, all participants reported their age, and so no participants needed to be excluded on this basis.

**Table 2:** **Best model summary for Experiment I**.

**Formula: correct** ~ **item in training** + **age** + **(1** + **item in training** | **participant)**				
	**Estimate**	**Std. Error**	***z*****-value**	**Pr(**>|**z**|**)**
(Intercept)	−2.17	1.17	−1.85	0.06
Item in training = TRUE	1.14	0.33	3.49	0.00
Age	0.11	0.04	3.05	0.00

The model shows that participants are more likely to pick the correct suffix in test if they have seen the item in training. Age is a significant predictor—older participants are more likely to give correct answers. This effect is not strong compared to presence in training, but it is robust and remains even if we remove margin values.

### 4.6. Discussion

The results of Experiment I confirm that, within the current design, many people are able to accurately learn a morpho-phonological pattern they were trained on. They are able to ascertain the triggering linguistic context and choose the appropriate answer. They are also able to generalize this pattern to items not seen during training. This remains true despite the relatively low number of training items with which the cue was presented. However, performance is somewhat better for test items previously seen in training.

Test behavior follows training behavior closely. Participants who finish training earlier are more likely to have a high accuracy in test.

Figure 4 shows that average participant accuracy in test has a bimodal distribution. Those participants whose accuracy is below the overall mean (at 0.74) have means clustered around 0.5 (equivalent to chance), while participants above the overall mean have means clustered toward 1 (equivalent to a perfect score). Based on this difference we can divide participants into “good learners” and “poor learners.” This grouping is supported by the training data. If we compare training length for the “good learner” participants (those with mean above the overall mean in test) and the “poor learners,” we find that the former finish training faster (as supported by a Wilcoxon rank sum test, *W* = 56, *p* < 0.001). Note that there are 19 good learners and 26 poor learners, suggesting that the task is relatively hard (with a “passing rate” of 42%). These results may be compared to those in Becker et al. (2011), an experimental study of nonce words in Turkish in which vocalic cues to an alternation were found to be less learnable than a consonantal cue. Our results also show that many participants have difficulties learning a vocalic cue to an alternation.

The mean trial count of the “good learner” group in training is 32. That of the “poor learner” group is 51. Recall that training has 24 unique trials. If a participant does not find out the key to success in training (the stem vowel), and keeps guessing, but remembers every single guess and identifies it correctly afterwards, they will need about 36 trials to finish training on the average (since they have a 50% chance of guessing right in the first place, and only need to repeat half of the trials). If they keep guessing, they need 518 trials on the average. The mean of the “poor learner” group is clearly between these values, suggesting that some rote learning did take place for this group (no participant needed 518 trials to finish), but it was not entirely efficient.

The “good learner”/“poor learner” distinction is *post-hoc*. Although we expected individual variability in learning, we did not hypothesize beforehand that listeners would fall into two clusters, with rather few “intermediate learners” falling between the “poor” and “good” learners. One possible interpretation is that the good learners are people who became consciously aware of the relevant cue. Conscious learning, also described in the research literature as “explicit learning” is generally faster than unconscious, or implicit learning (Goujon et al., 2015). While the good learners recognize the contextual pattern and simply apply it to all new items, some poor learners seem to perform rote learning as they repeatedly see training items. They do learn the correct suffixed form for some specific items, as evidenced by the greater number of participants who perform above chance in seen items, but they are not successful in generalizing to new items. It is also possible, of course, that this distribution does not relate to an explicit/implicit learning distinction at all, but rather reflects the distribution of individual learner characteristics in our data-set. Brooks et al. (2016), for example, have shown that morphological learning and generalization varies across individuals, in a way that correlates with measures of non-verbal intelligence and general statistical learning abilities. What follows is that if key individual learner characteristics are bimodal, then the learner outcomes would also be bimodal.

The results of Experiment I give us indications on how participants proceed through a learning task based on a cue associated with a linguistic context. The decisive point is whether a participant learned the pattern, and if this does not happen in training, participants will mostly guess in test. A sizeable group, but still a minority, learned the general cue association pattern. In Experiment II, we look at a similar task that uses a non-linguistic context.

## 5. Experiment II

In this experiment, the cue is no longer related to the name used by the conversation partner—rather, it is the conversation partner itself.

### 5.1. Participants

One hundred and five participants were recruited through Amazon Mechanical Turk. 51 are women, 54 men. Mean participant age is 34 years, with a standard deviation of 9.62. 54 participants were assigned to the *view* condition, 51 to the socially relevant *gender* condition. Four participants were excluded for not following the instructions properly. Four participants were removed based on training speed. We report data from the remaining 97 participants. All participants are native speakers of American English. Each person was paid three dollars upon completion of the task.

### 5.2. Methods

Experiment II modifies Experiment I in one major way. The correct response no longer depends on the vowel of the prompt. Rather, it depends on the conversation partner, who was irrelevant in Experiment I. A non-linguistic contextual cue replaces the linguistic cue in learning a morpho-phonological pattern. The non-linguistic contextual cue is relatively basic. It is either who the conversation partner is or what physical orientation they have compared to the protagonist.

Experiment II has the same conversation partners as Experiment I, who, again, can each be seen in two different ways. This creates two groupings. One grouping, *gender*, is the identity of the conversation partner—who is either male or female. The other, *view*, is the spatial orientation of the conversation partner. The aim of this design is to teach naming patterns in conjunction with the *contextual cue* provided by the grouping. The images used can be seen in Figure 2. Learning the “view” cue requires participants to notice that changes in language use are correlated with changes in the direction the partner is facing. Learning the “gender” cue requires the participants to notice that changes in language use are correlated with changes in the speaker.

Who your conversation partner is has a huge effect on linguistic category learning. Listeners are able to keep track of information coming from two different speakers, adapt to new speakers, and recognize the difference between across-speaker and within-speaker variation and weigh them differently (Horton and Gerrig, 2002, 2005; Kraljic et al., 2008a). Perceived speaker gender is an especially robust cue (Johnson et al., 1999).

In contrast, conversation partner spatial orientation is much less salient as a social-indexical cue. People learn both deictic expressions (denoting spatial relations, such as “here” and “that”) and words with implicit spatial relations (such as “wide” or “tall”) easily, since these are frequent forms of every language. Variation between deictic expressions, however, does not typically carry social meaning.

Note that, if our participants in this task learn the association of the linguistic pattern with conversation partner, we have no way of knowing whether they are imputing a person-specific pattern, or a more general distinction based on person gender. As the most salient difference between the two partners is the gender difference, we here refer to the cue as a *gender* cue. Whether the learned cue is identity or gender can not be established from the design of Experiment II. However we will explicitly test the degree to which the learning to generalized to other speakers on the basis of gender in later experiments.

The game, then, has four conversation partner images. Each occurs once with each item in training. Again, targets are presented in a random order to each participant, and the participant has to give a correct answer for every target in order to move to the next target. In the test phase, targets are presented, again, in random order. No feedback is given. Training consists of six items, so it has 4 × 6 = 24 targets in total. The test contains these six items, and six items unseen in training, presented with each of the four conversation partners, so it has 4 × 12 = 48 targets in total.

The nonce language we used is similar to Experiment I, except that the vowel harmony pattern is absent. Instead, we used the five English vowel letters to make stimuli maximally distinct. The list of stimuli used in Experiment II can be seen in Table 3. The same principles guided stimuli selection as in Experiment I. Since stem vowel is no longer relevant, we used the five English vowel letters to make the syllables more distinct. For each participant, two syllables are randomly selected as suffixes (marking conversation partner gender or spatial orientation, depending on the condition) while the rest are randomly assigned as item names.

**Table 3:** **Stimuli set, Experiment II**.

fek	rik	wuk	fal
pel	ril	tol	rul
wan	fen	wun	tas
fis	tos		

There are four conversation partner images in the experiment, and two suffixes. Each suffix corresponds to two conversation partner images. The *across-subject* factor of Experiment I is the grouping of the conversation partner images. In the *gender* condition, the correct suffix (and, consequently, the correct response) is *cued* by the identity/gender of the conversation partner. In the *view* condition, the correct suffix (and so the correct response) is cued by the conversation partner's orientation (facing outwards or facing left).. The *within-subject* factor is whether a test item was seen in training.

### 5.3. Hypotheses

Experiment II looks at the association of a morpho-phonological pattern and a non-linguistic context. We had three hypotheses for Experiment II: (i) Participants would learn the diminutive pattern and extend it to new items in the *gender* condition (ii) learning and extension would be poorer in the *view* condition (iii) participants would be more likely to assign the correct pattern to items in the test phase if they have *seen* them in the training phase. We also evaluated participant age and gender as predictors of performance.

### 5.4. Results

We find that the pattern is indeed easier to learn with the *gender* condition. Unlike in Experiment I, item presence in training has no effect on response accuracy.

We use two measures of participant performance. In the training phase, we look at the number of trials it takes a participant to finish the experiment. This number provides information about the difficulty of learning in training and how much attention the participant pays to the task—this is why we use it as our main exclusion criterion. Participant responses in the test phase tell us how much they remember training and how easily they extend the pattern to new items and conversation partners.

Training takes longer (in terms of trial counts) in Experiment II (*m* = 66, *sd* = 25) than in Experiment I (*m* = 42, *sd* = 18) (a significant difference according to a Wilcoxon rank sum test, *W* = 3450, *p* < 0.001).

**Figure 5:**
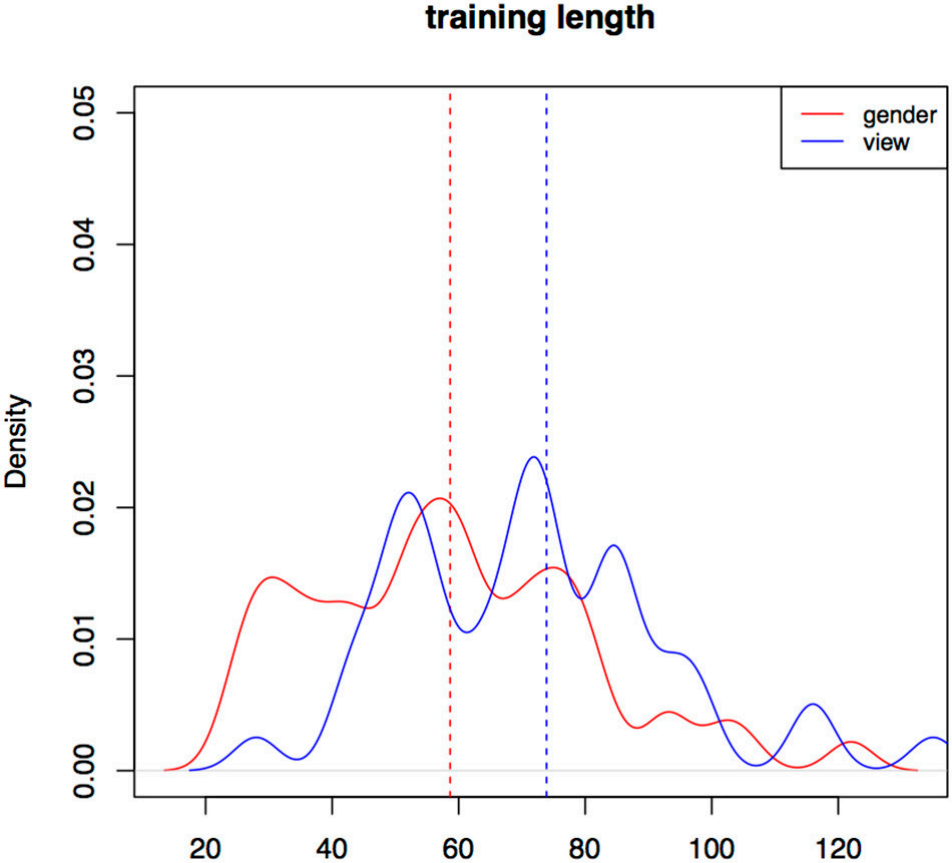
**Distributions of training trial counts for the two conditions for all participants, Experiment II**.

In Experiment II, participant training trial count is longer in the *view* condition (*m* = 74, *sd* = 27) than in the *gender* one (*m* = 59, *sd* = 22, *W* = 1565, *p* < 0.01). Training with the *gender* cue in Experiment II is still significantly longer than training in Experiment I. Figure 5 is a kernel density plot of training trial count for individual participants grouped by the two conditions. Mean trial count is shorter in both conditions than what we would expect for random behavior. Trial count is the number of trials it takes a participant to finish training. The smoothing bandwidth and the y axis are held constant for all density plots in this paper to aid comparison.

**Figure 6:**
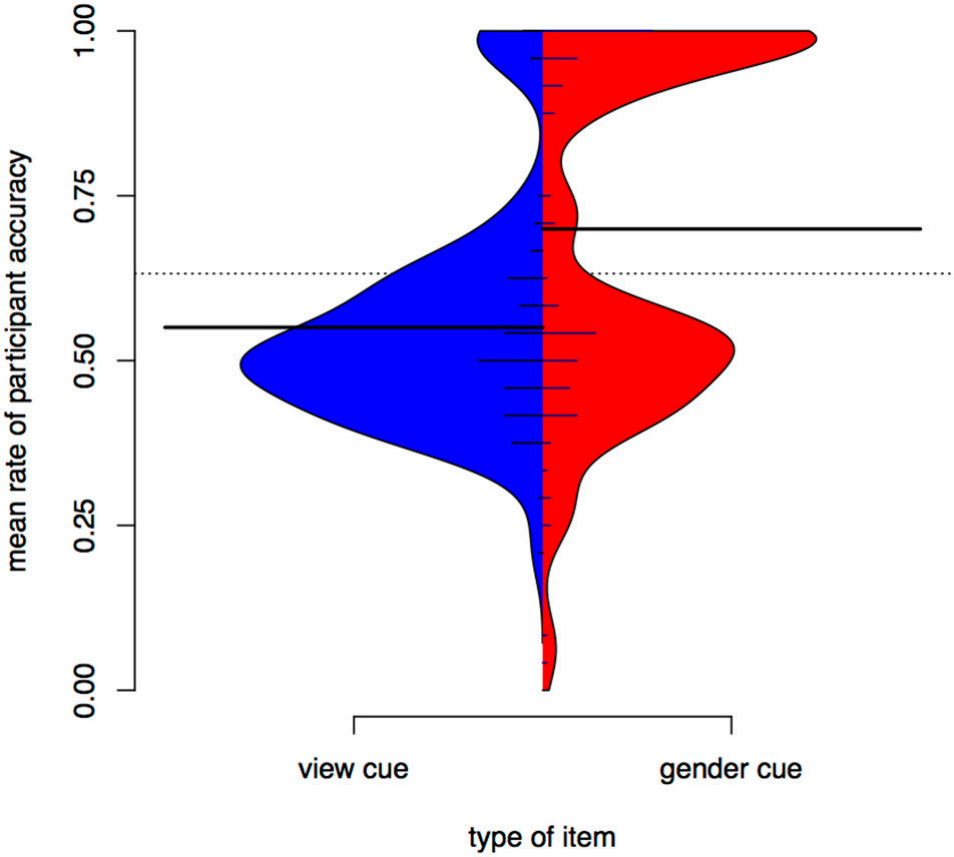
**Distributions of participant responses with different contextual cues, Experiment II**. Black horizontal bars show the mean accuracy for each set of items. The dotted line shows the overall mean. Small horizontal lines show individual values; longer if multiple individuals have the same average.

Figure 6 is a bean plot of participant responses, contrasting the *gender* condition and the *view* condition. For the *view* cue, most participants have a mean around 0.5—they are effectively guessing in test. In contrast, a sizeable proportion of participants has high accuracy for the *gender* cue. The bimodal structure of the *view* distribution strongly resembles the distribution of participant results in Experiment I for the unseen items.

We stepwise fit a binomial mixed-effects regression model on the test data, using response to individual items (*correct* or *not correct*) as an outcome variable and cue type (*gender* or *view*), *item presence in training*, and participant age and gender as predictors, with a participant random intercept. The summary for the best model can be seen in Table 4. Since age was a significant predictor, this model excludes 2 out of 97 participants who had no age data available. Model fitting in Experiment II is similar to Experiment I, we start with the most complex regression model and remove predictors one after the other until we reach the best fit. We test for all interactions of our terms.

**Table 4:** **Best model summary for Experiment II**.

**Formula: correct** ~ **age** + **cue type** + **(1** | **participant)**				
	**Estimate**	**Std. Error**	***z*****-value**	**Pr(**>|**z**|**)**
(Intercept)	−1.51	0.63	−2.40	0.02
Age	0.06	0.02	3.21	0.00
Cue type = gender	1.09	0.34	3.20	0.00

The model shows that participants who are trained on the *gender* cue have much higher accuracy in test. Unlike in Experiment I, item presence in training is irrelevant—participant accuracy remains the same with previously seen and unseen items. Age is a significant predictor of test accuracy: older participants are more accurate.

### 5.5. Discussion

Cue type is a strong and independent predictor of test accuracy in Experiment II. Participants trained with the *gender* cue have a much higher test accuracy, echoing results in the contextual learning of phonetic categories. Item presence in training does not affect test accuracy.

The Somers' Dxy Rank Correlation between test response accuracy and training trial count is modest (0.37). This is probably because participants show two types of behavior, much as in Experiment I. As we speculated above, some participants may have explicitly recognized the the context-pattern association, while others did not.

If we group participants with mean test accuracy above the overall mean as “good learners” and those below the overall mean as “poor learners,” we find that good learners finish training in significantly fewer trials (*W* = 484, *p* < 0.001).

If we look at the distribution of good learners across cue type, we find that most good learners are to be found in the *gender* condition (cf. Table 5). This tabulation supports the results of the regression analysis: the context-pattern association is easier to recognize for the *gender* cue than for the *view* cue.

**Table 5:** **Good learners and poor learners across cue type, Experiment II**.

	**View cue**	**Gender cue**
Good learner	5	25
Poor learner	40	27

When we compare Experiment II with Experiment I, we see that learning the non-linguistic cue is harder than learning the linguistic cue. As we note above, training takes longer. This remains true if we compare the *gender* cue with the linguistic cue in Experiment I (learning the linguistic cue takes significantly fewer trials, *W* = 639, *p* < 0.001). An important difference between Experiment I and Experiment II, however, is that the linguistic cue is learned through exposure to a range of linguistic items, but the gender cue is learned through a contrast between just two people. A number of studies have shown that repetition and variability of context leads to improved learning (Gómez, 2002; Rost and McMurray, 2009). We cannot therefore directly compare the learnability of the linguistic and the social cue from these experiments alone.

**Figure 7:**
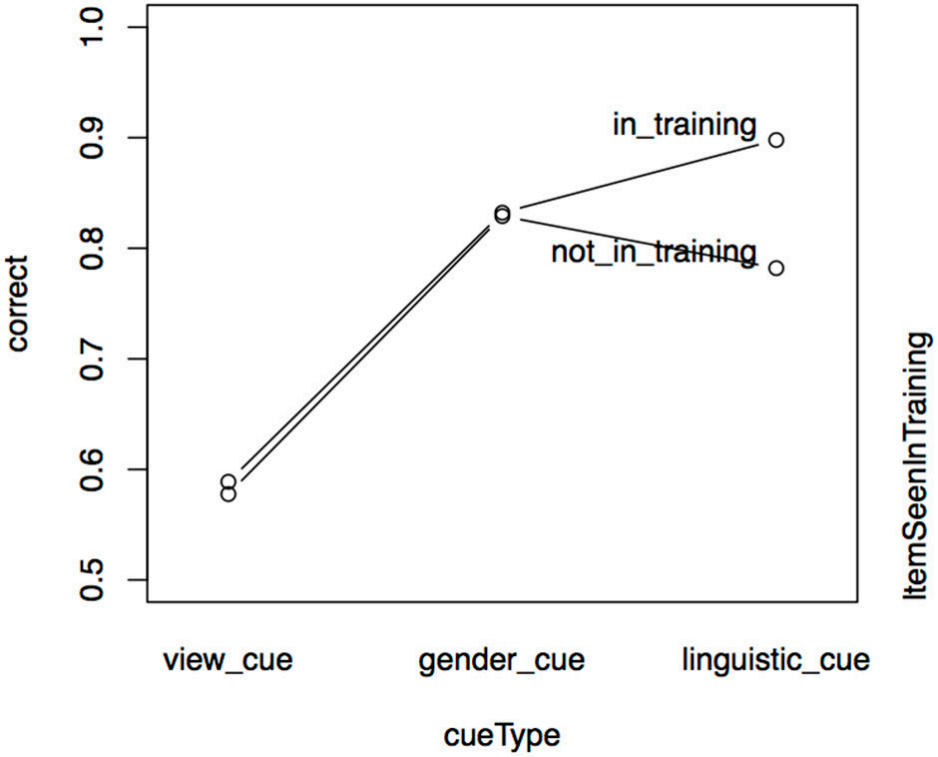
**Effect of cue type (view cue, gender cue, or linguistic cue) and item presence in training in the test data for Experiments I and II**. Results of mixed effects model on combined data set.

Test accuracy for the *gender* cue in Experiment II is not worse overall than test accuracy in Experiment I. There is, however, an important difference in relation to item presence in training, which is significant in Experiment I but not in Experiment II. We merged the data from Experiments I and II and performed a binomial mixed-effects regression analysis, using the interaction of *item presence in training* and *cue type* (view, gender, linguistic) as predictors, with a participant random intercept. Effect sizes can be seen in Figure 7.

For the two contextual cues tested in Experiment II, item presence in training is not relevant. For the linguistic cue in Experiment I, participants are better at recalling names for items they have seen in training. This result could indicate that rote-based learning of items is relevant for the linguistic cue, but less so for the contextual cues (even for the *gender* cue, where a substantial amount of learning takes place). Note, however, that the role of the stem is different in the two experiments. In Experiment I, the participant must attend to the different stems in order to select the correct suffix. In contrast to Experiment I, the key to success in Experiment II is paying attention to the social context. The available responses always share the stem with the prompt word, which is irrelevant in relation to success on the task. This fact could explain the differing outcomes.

It remains clear that, in Experiment II, most learning happens with the socially salient, interpretable cue, *gender*—the identity of the conversation partner. The notion of social salience afforded by this experiment, however, is very narrow—it entails a distinction between two specific conversation partners (a woman and a man) as opposed to their position in space.

We have referred to the two cues as *view* vs. *gender*, assuming it is very likely that participants rely on the visible gender difference between the conversation partners in making their decisions. In Experiment II it is impossible to know whether participants are performing a categorization based on speaker gender, or simply associating the cue with the particular speakers. In Experiment III, we therefore continue exploring non-linguistic contexts, by more explicitly testing whether the associations learned in this type of experiment are extended to other partners, on the basis of conversation partner gender.

## 6. Experiment III

In this experiment, we look at whether participants generalize from the learning process we have seen in Experiment II, by extending the contextual cue to new conversation partners on the basis of gender.

### 6.1. Participants

The experiment was hosted on Amazon Mechanical Turk. 101 people participated in the experiment. 57 are women, 44 men. 50 are in the *gender* condition, 51 in the *view* one. Mean age is 32 years, with a standard deviation of 10.83. Four were excluded from the analysis based on training length. We report data from the remaining 97 participants. All are native speakers of American English. Participants were paid three dollars upon completion of the task.

### 6.2. Methods

Experiment III was designed to replicate the results of Experiment II, and in addition it investigates whether participants are able to generalize the contextual cue to new conversation partners in the test phase. It was identical to Experiment II except for the fact that Experiment III has eight conversation partners instead of four. Four conversation partners are present in training and test (just like in Experiment II) and four conversation partners are only present in test. Both previously seen and novel items are presented with previously seen and novel conversation partners in test, making the test twice as long as in Experiment II.

Experiment III uses all the conversation partner images in Figure 2. Conversation partners are grouped according to a *gender* attribute, as well as a *perspective (view)* one, their spatial orientation. We used an adult/child distinction for conversation partner images that are present in training vs. unique to the test. The reason for this is that we wanted to keep the two conversation partner categories distinct visually. Some higher level knowledge is needed to realize that an adult and a child share the same gender. In contrast, two adult images of the same gender could have been matched based on visual similarity only.

The experiment has two across-subject factors. Half the participants have to learn the relevant cue (*gender*), and half of them the accidental cue (*view*). Also, half the participants are trained with children, and the other half with adults, creating four different training groups.

### 6.3. Hypotheses

We evaluated four hypotheses for Experiment III: (i) Participants would learn the diminutive pattern and extend it to new items and new conversation partners in the *gender* condition, (ii) The diminutive pattern would be easier to learn if it is associated with the *gender* cue than with the *view* cue (iii) Participants would be better able to assign the correct pattern to items in the test phase if they have seen them in the training phase, (iv) participants would be better able to assign the correct pattern to conversation partners that they had seen in the training phase. We also evaluated age and gender as predictors.

### 6.4. Results

**Figure 8:**
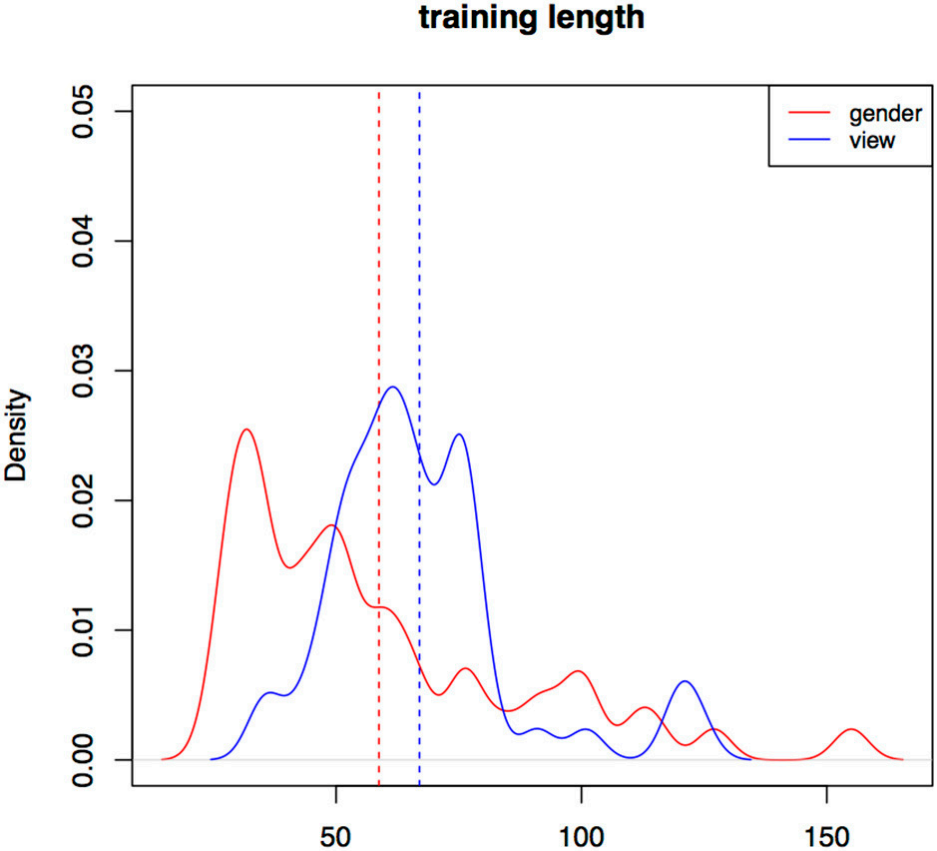
**Distributions of training trial counts for the two conditions for all participants, Experiment III**.

Participants finish training much faster than a player would at random. On average, it takes participants longer to finish training in the *view* condition than in the *gender* condition (a significant difference according to a Wilcoxon rank sum test, *W* = 1556, *p* < 0.01). Figure 8 is a density plot of training length for individual participants grouped by the two conditions. Training trial count in Experiment III is not significantly different from Experiment II.

**Figure 9:**
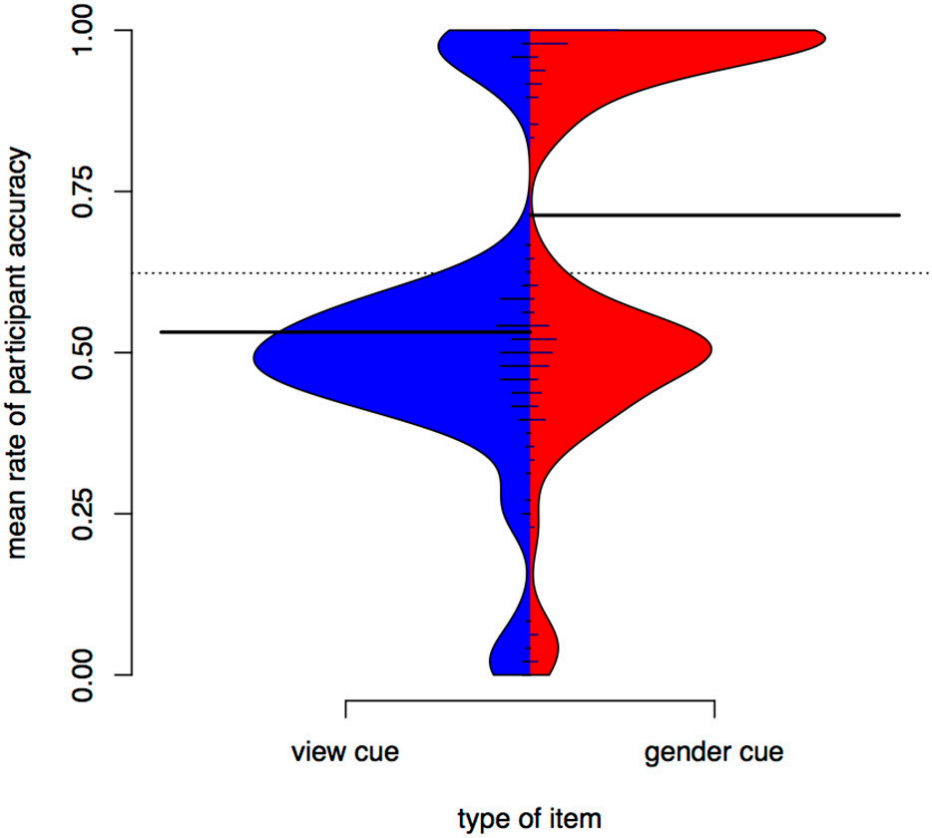
**Distributions of participant responses on test items in the ***view*** and ***gender*** conditions, Experiment III**. Black horizontal bars show the mean accuracy for each condition. The dotted line shows the overall mean. Small horizontal lines show individual values; longer if multiple individuals have the same average.

Figure 9 shows a bean plot of participant test responses for the *gender* condition and for the *view* condition. Mean accuracy is much higher for the *gender* condition.

We stepwise fit a binomial mixed-effects regression model on the test data, using response to individual items (*correct* or *not correct*) as an outcome variable and the interaction of cue type (*gender* or *view*) and item *presence in training*, conversation partner *presence in training*, conversation partner *type* in training (*children* or *adults*), and participant age and gender as predictors, with a participant random intercept. Response accuracy is predicted by cue type. It does not depend on familiarity with items or conversation partners. Accuracy does not improve significantly with age, or differ by participant gender. The summary of the best model can be seen in Table 6.

**Table 6:** **Best model summary, Experiment III**.

**Formula: correct** ~ **cue type** + **(1** | **participant)**				
	**Estimate**	**Std. Error**	***z*****-value**	**Pr(**>|**z**|**)**
(Intercept)	0.28	0.31	0.90	0.37
Cue type = gender	1.68	0.44	3.81	0.00

### 6.5. Discussion

The results of Experiment III support the results of Experiment II, and show that the learning generalizes to other partners. Even when exposed to just one person in the training, participants extend this learning to others, on the basis of the person's *gender*.

While half the participants are trained with children and the other half with adults, this makes no difference in test accuracy.

This further supports our assumption that the perceptual difference between our conversation partner images is far less relevant than their socially salient grouping characteristics.

As in the previous two experiments, participant mean test accuracy ratings show a clear bimodal distribution. We can group participants as good learners or poor learners, according to whether their test mean is above or below the overall mean. If we tabulate good learners across cue type, we find that the *gender* cue is easier to learn. This can be seen in Table 7.

**Table 7:** **“Good” learners across cue type, Experiment III**.

	**View cue**	**Gender cue**
Good learners	8	25
Poor learners	41	23

The results of Experiment III are very similar to Experiment II. The main difference is that, in Experiment III, we have evidence that participants clearly rely on a more abstract context to establish generalizations. If they recognize conversation partner gender as the contextual cue, they are able to interpret it generally. They are able to learn this cue with adults and extend it to children and vice versa. This is comparable to the recognition of phonetic categories in stereotypical male and female voices. The huge difference is, however, that this distinction is both much more abstract (relying on a distinction in diminutive use) and simpler (a single difference in suffixes as opposed to a complex envelope of distinction between stereotypical male and female voices). This grants additional power to our socially salient distinction, which is generalized to differences between stereotypically male and female characters. This distinction, trained with only one instance of each gender, is straightforwardly generalized to a new instance (from a woman to a girl, etc.).

Now that we have established that learning based on just one person is extended to another person of the same gender in the test, this substantiates the choice of *gender* (rather than identity) as the most appropriate label to use for the person-based cue.

Note that the item presence in training is not a significant predictor of test accuracy in either Experiment II or III. This suggests that participants completely disregard the prompt word form and focus on the suffix and the associated context (if they focus on anything at all). The design of these experiments, however, does not allow us to explicitly test whether participants pay attention to the suffix vs. the stem and how this relates to training performance. Experiment IV addresses this question.

## 7. Experiment IV

In this experiment, we return to the learning process in Experiment II and look at the relative importance of our various cues by offering participants two test choices that are both “wrong,” in different ways.

### 7.1. Participants

The experiment was hosted on Amazon Mechanical Turk. 80 people participated in the experiment. 46 are women, 34 men. 40 are in the *gender* condition, 40 in the *view* one. Mean age is 32 years, with a standard deviation of 9.99. Two participants were excluded from the reported data based on training length. We report data from the remaining 78 participants. All participants are native speakers of American English. Participants were paid three dollars upon completion of the task.

### 7.2. Methods

Experiment IV uses the adult *woman* and *man* conversation partners in *front* and *side* view.

For Experiment IV, as for Experiment II, context determines the correct response during the training phase. Each target has two possible responses. One has the suffix associated with the present context, the other has the suffix associated with the absent context. So, if the context is *gender* the participant must choose the response with the suffix that matches the gender of the conversation partner on screen. The stem of the two available responses is always the same, the name of the query, which is also visible on the screen.

The test phase of Experiment IV differs from Experiment II in two respects. First, during the test phase, participants are only exposed to previously seen items, no novel items are presented. And second, the query and the prompt name are no longer visible on the screen. One possible response for the target has the stem which is the name of the query of the target (as seen in training) but a context-inappropriate suffix (this is a choice present in the previous experiments). The other possible response has the correct suffix, but it has a stem that is not the name of the query of the target (as seen in training). Both answers are wrong (compared to training), but for different reasons. One has the correct prompt name, one the correct suffixation pattern, but neither has both. Table 8 gives an example.

**Table 8:** **Example stimuli, Experiment II vs. Experiment IV**.

	**Training**		**Test**	
Exp II	stem	tas	stem	tas
	stem+correct suffix	tasrul	stem+correct suffix	tasrul
	stem+incorrect suffix	taspel	stem+incorrect suffix	taspel
Exp IV	stem	tas	(stem not visible)	
	stem+correct suffix	tasrul	incorrect stem + correct suffix	fenrul
	stem+incorrect suffix	taspel	correct stem + incorrect suffix	taspel

In the training phase, the stimuli were generated from the same pool as in Experiment III. For each participant, two syllables are assigned as suffixes. Six syllables are assigned as item names. In test, the “wrong conversation partner” answer was generated using the prompt name and the wrong suffix. The “wrong prompt name” was generated using a different, randomly assigned prompt name and the correct suffix. This means that the wrong stems were different for the same item across test trials.

Picking the response with the correct stem (the name of the query in training) but the wrong suffix (the one that belongs to the other cue) means that, during training, participants pay more attention to the entity they name than the context. Picking the response with the correct suffix (the one that belongs to the present cue) but the wrong stem (not the name of the query) means that, during training, participants pay more attention to the context than the entity they name. The naming task in Experiment II and III derive the name from the query image and the conversation partner, and the naming task in Experiment IV allows directly comparing their degree of relevance.

### 7.3. Hypotheses

We had two hypotheses for Experiment IV: (i) as in the previous experiments, participants would finish training faster in the *gender* condition than in the *view* condition. (ii) Participants would be more likely to focus on the suffix in the *gender* than in the *view* condition; as seen in experiments II-III, the *gender* cue contributes more to learning success, and hence it is likely easier to recognize and learn.

### 7.4. Results

The overall training duration of Experiment IV is not significantly different from that of Experiment III or Experiment II. As in Experiment II, training in the *gender* condition is significantly shorter than in the *view* one (*W* = 1023, *p* < 0.01, using a Wilcoxon rank sum test).

In the test phase, overall, participants pick the answer containing the correct suffix significantly more often than the answer with the correct stem (the “original” name) (59% of the time).

During test, participants in the *view* condition pick the correct stem overwhelmingly more (76% of the time) than in the *gender* condition (41% of the time). In the *gender* condition, the correct suffix is preferred more often (59% of the time).

**Figure 10:**
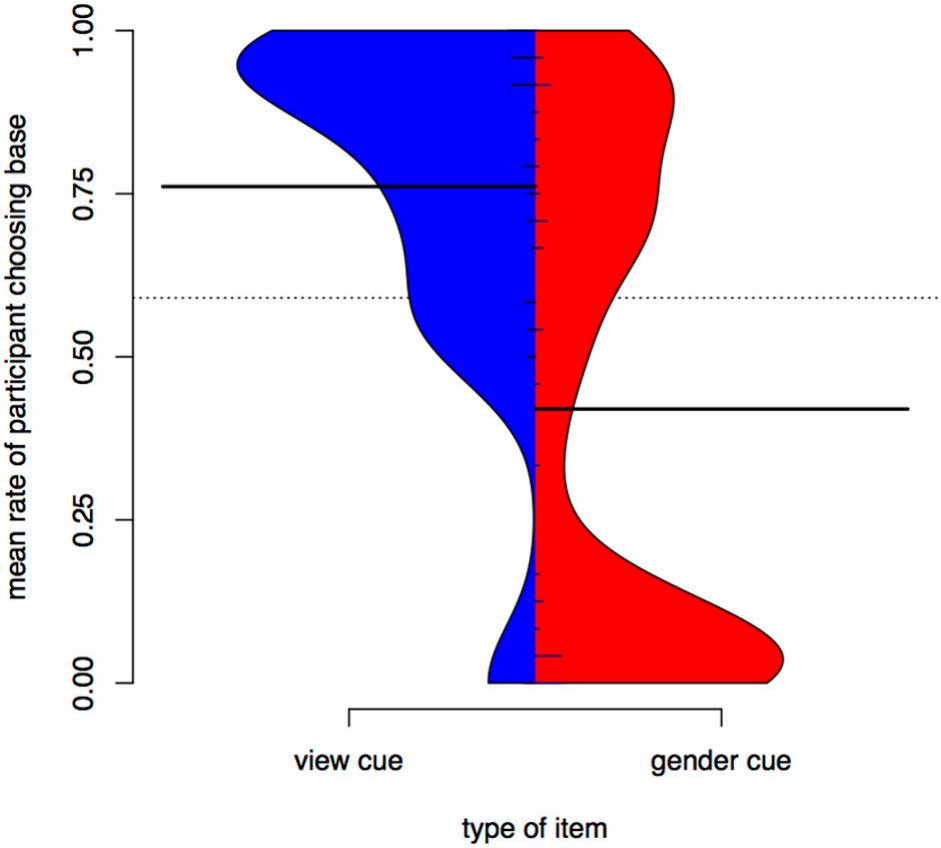
**Distributions of participants picking right stem plus wrong suffix (1) vs. wrong stem plus right suffix (0) in the two conditions, Experiment IV**. Black horizontal bars show the mean rate of “stem” preference for each condition. The dotted line shows the overall mean. Small horizontal lines show individual values; longer if multiple individuals have the same average.

Figure 10 shows the degree to which participants pick the stem (1) or the suffix (0), that is, the preference for the *stem* with the *view* cue (left) and the *gender* cue (right).

We stepwise fit a binomial mixed-effects regression model on the test results using “picked correct stem” (as opposed to “picked correct suffix”) as outcome variable and condition (*gender* or *view*) and participant age and gender as predictors, with a participant random intercept. The summary of the best model can be seen in Table 9. The only significant predictor is the condition, with the *view* cue leading to a stronger preference for the stem than the *gender* cue.

**Table 9:** **Best model summary, Experiment IV**.

**Formula: correct stem** ~ **cue type** + **(1** | **participant)**				
	**Estimate**	**Std. Error**	***z*****-value**	**Pr(**>|**z**|**)**
(Intercept)	1.86	0.44	4.27	0.00
Cue type = gender	−2.81	0.62	−4.54	0.00

### 7.5. Discussion

In the *view* condition, participants overwhelmingly focus on the stem of the response, rather than the suffix. This suggests that, in the *gender* condition, the suffix is much easier to learn than in the *view* condition. This is also the outcome for Experiments II & III: accuracy for the *view* condition is not much higher than chance. Some participants learn the *view* cue, but many fewer than the *gender* cue.

**Figure 11:**
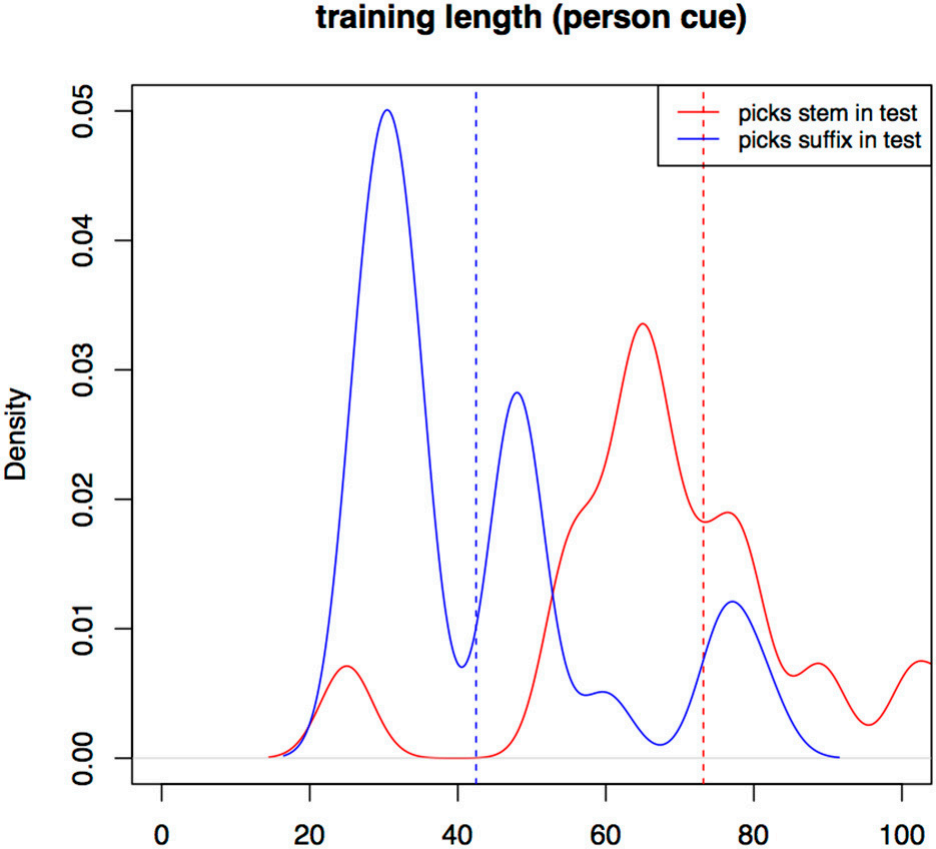
**Distributions of training trial counts for “object” and “people” participants, Experiment IV**.

The *gender* condition of Experiment IV is more interesting. The tight answer ratio (59 vs. 41%) indicates that participants can rely on either—there are “object” people and “people” people. This difference does not vary with participant age or gender. We can infer more about being an “object” or a “people” person as a learning strategy if we look at training performance for these two groups. Figure 11 shows training trial counts for participants who overwhelmingly go for the stem or the suffix in test.

People who go on to pick the suffix in test are much faster to finish training than people who go on to pick the stem. We can interpret training and test performance in Experiment IV as results of either of two learning strategies. “Object” people focus on the stem, therefore take a while to finish the training, and overwhelmingly pick the stem in the test. “People” people focus on the suffix, finish training much faster, and pick the suffix in the test. We should note that “object” participants in the *gender* condition are as slow in training as participants in the *view* condition.

In the *gender* condition, as in the analogous conditions of experiments II-III, both the linguistic context and the non-linguistic context of the morphological pattern (the prompt name and the conversation partner's gender/identity) are readily available. A group of participants are able to pin down the relevant factor in variation, namely, the conversation partner, and pick their responses accordingly. Others remain “distracted” by the prompt name. In the *view* condition, the non-linguistic context is barely if at all accessible—consequently, all participants focus on the linguistic context.

We use the word “focus” to refer both the participant's attention (what part of the frame they pay attention to) and the participant's weighing of the cues (how much importance they attribute to any cue; that is, any part of the frame that changes from trial to trial). These cannot be separated in our analysis, but likely together lead to the observed dichotomized participant behavior, dividing successful and poor learners in the experiment.

We now have strong reasons to believe that a robust and salient non-linguistic context is easier to learn than a less salient one. The generality of these findings, however, is somewhat compromised by the fact that we have thus far only looked at one morphological pattern, the diminutive, which is both highly variable in English and which has strong associations with gender in many languages (Jurafsky, 1993/2012). In order to make our findings more robust, we repeated Experiment III using the plural instead of the diminutive as the iconic relationship between prompts and targets. The main question was whether participant accuracy changes with visual stimuli cueing the plural replacing diminutive stimuli.

## 8. Experiment V

In this experiment, participants work with an artificial language that is based on a different iconic relationship, the plural instead of the diminutive.

### 8.1. Participants

The experiment was hosted on Amazon Mechanical Turk^2^. 89 people participated in the experiment. 50 are women, 39 men. 46 are in the *gender* condition, 43 in the *view* one. Mean age is 37 years, with a standard deviation of 15.47. All are native speakers of American English. Participants were paid three dollars upon completion of the task. We excluded 4 participants from the analysis based on training speed. We report data from the remaining 85 participants.

### 8.2. Methods

Experiment V is identical to Experiment III except for the prompt and target images. Experiment III, like all previous experiments, used a normal sized item and a diminutive item as the pair of pictures. The instructions told the participant to identify the name of the small item based on the larger item. In Experiment V, by contrast, each query picture displays an item and each target picture displays three of the same item. The instructions tell the participant to identify “the plural, the word for multiple instances of the same item.” Otherwise instructions are unchanged. The goal of Experiment V is to determine whether the results of Experiment III generalize to a morphological process (pluralization) that is highly general and productive in English and many other languages.

Experiment V uses all conversation partners in *front* and *side* view.

### 8.3. Hypotheses

Our hypothesis was that the patterns that we had previously observed would generalize beyond the particular case of the diminutive. We therefore evaluated the same hypotheses for Experiment V as for Experiment III. Based on the results of Experiment III, we expected that participants would learn the plural pattern and extend it to new items and new conversational partners. We expected learning to be more successful in the *gender* condition than in the *view* condition. We expected no advantage for seen items or partners, and we also looked for effects of participant age and gender.

### 8.4. Results

**Figure 12:**
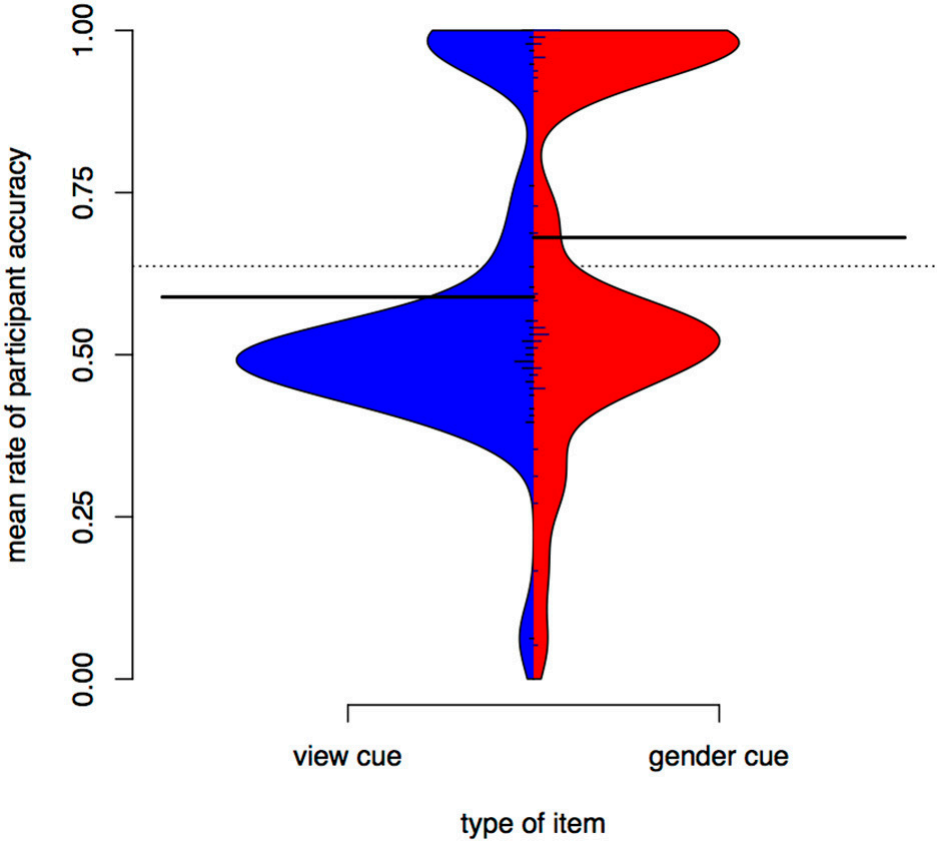
**Distributions of participant responses on test items in the ***view*** and ***gender*** conditions, Experiment V**. Black horizontal bars show the mean accuracy for each condition. The dotted line shows the overall mean. Small horizontal lines show individual values; longer if multiple individuals have the same average.

Here, we first look at Experiment V by itself and then together with Experiment III. Cue type has no effect on training speed in Experiment V. The mean rate of participant accuracy in test can be seen in Figure 12. We fit a mixed-effects binomial regression model on the test data using item and conversation partner presence in training, type of cue, and participant age and gender as predictors, with a participant random intercept. The model summary can be seen in Table 10. The only predictor that shows any effect is cue type. The effect size is above the level of statistical significance. This result is similar to what we see in Experiment III, even though the effect is weaker in test—and absent in training.

**Table 10:** **Best model summary, Experiment V**.

**Formula: correct** ~ **cue type** + **(1** | **participant)**				
	**Estimate**	**Std. Error**	***z*****-value**	**Pr(**>|**z**|**)**
(Intercept)	0.77	0.33	2.33	0.02
Cue type = gender	0.84	0.46	1.83	0.07

The only way to tell whether the experiments differ from each other significantly is to use statistical tests on the joint data from the two experiments.

Training in Experiment V does not differ significantly in length from training in Experiment III.

We merged the two datasets and stepwise fit a mixed-effects binomial regression model on the combined test data using item and conversation partner presence in training, type of cue, type of pattern (*diminutive* or *plural*), and participant age and gender as predictors, with a participant random intercept. The plural dataset patterns essentially the same as the diminutive dataset. *Cue type* is a significant predictor of test accuracy. Participant age and gender and item presence in training are not significant. The type of pattern (diminutive/plural) does not affect test accuracy significantly. The summary of the best model of the merged test data can be seen in Table 11.

**Table 11:** **Best model summary, Experiments III and V**.

**Formula: correct** ~ **cue type + (1** | **participant)**				
	**Estimate**	**Std. Error**	***z*****-value**	**Pr(**>|**z**|**)**
(Intercept)	0.50	0.23	2.22	0.03
Cue type = gender	1.29	0.32	4.03	0.00

### 8.5. Discussion

Experiment V shows that the learning difference between the socially salient cue and the irrelevant cue persists when these cues are tied to a different morphological pattern, the plural. This adds further robustness to this distinction.

When we look at the experiment in itself, the effect of the gender cue is weaker than in other experiments, e.g., Experiment III. It is also above the generally accepted threshold of statistical significance. However, the statistical analysis of the two datasets together indicates that this difference is not statistically significant. The joint analysis gives us no ground to reject the null hypothesis that the plural does not differ from the diminutive. In general, finding significance levels for differences in statistical significance is difficult and would require a study considerably exceeding our scope at present. If future work establishes that socio-indexical associations for plural patterns are indeed more difficult to learn than for diminutive patterns, the reasons for this difference would be of considerable interest. Potential factors could include adult differences in the adaptability of the derivational vs. the inflectional morphology, and pre-existing associations between the diminutive and social attributes of age, gender, or status (as described in Jurafsky, 1993/2012 as well as Kruisinga, 1942 cited by Bauer, 1997). For English, a further aspect is that the language has a number of competing diminutive suffixation patterns (such as *-ling, -ly, -ie*, etc), but only one broad, productive plural pattern.

In experiments II, III, and V, it is only with the salient cue that participants show a large degree of learning. However, only about half the participants exposed to the salient cue show high accuracy in test, while the other half of this group resorts to guessing, much like participants learning the non-salient cue. In Experiment IV we looked at learning strategies and proposed that, when both types of information are accessible, some participants will focus on the linguistic context (the prompt), and others at the non-linguistic context (the conversation partner). What remains unclear is whether participants make a by and large random choice at the beginning to focus on either context and then remain with it, or whether the effect of the non-linguistic context can be increased by expanding training. This is an especially relevant question given that item presence in training does not affect test accuracy, suggesting that the recognition of the relevant context is far more important than exposure to the specific training items. We look at this question in Experiment VI.

Another important question arising from our results across experiments I-V is the role of individual participant characteristics. We evaluated age and gender as individual predictors, with mixed results. These participant characteristics were not controlled in the participant recruitment procedure, and different experiments enrolled slightly different age and gender distributions.

In order to obtain more statistical power to look at these participant effects, we combined the test data for experiments II, III, and V, which have the same training size, the same test setup, and the same cue differences (*gender* and *view*). The pattern is either the diminutive or the plural. Those are also comparable. Each experiment has items in the test phase that were seen in training as well as new items. We fit a binomial mixed-effects regression model on the combined test data for participants with age available—273 participants. The outcome variable is response accuracy, the predictors are participant age and gender, as well as cue type and item presence in training, with a participant random intercept. The best model has age and cue type as significant predictors. The model summary can be seen in Table 12. Older participants are more accurate overall, and responses to socially salient cues are much more likely to be correct. Cue type is a much stronger predictor than age. Participant gender is not a significant predictor. Similarly, inspection of the training data reveals a significant age effect, with older participants completing the training in significantly fewer trials.

**Table 12:** **Best model summary, Experiments II, III, and V**.

**Formula: correct** ~ **age** + **cue type** + **(1** | **participant)**				
	**Estimate**	**Std. Error**	***z*****-value**	**Pr(**>|**z**|**)**
(Intercept)	−0.51	0.43	−1.20	0.23
Cue type = gender	1.23	0.25	5.01	0.00
Age	0.03	0.01	2.45	0.01

The result shows that older participants are doing better with the socio-indexical learning. The effect, however, is not very strong.

## 9. Experiment VI

In this experiment, participants undergo an extended training phase. Extended training allows us to explore whether participants can modify their focus of attention based on feedback during training.

### 9.1. Participants

The experiment was hosted on Amazon Mechanical Turk. 100 people participated in the experiment. 55 are women, 45 men. 58 are in a *gender* condition. 42 participants are in the *view* condition. Mean age is 35 years, with a standard deviation of 10.98. Four participants were excluded based on training length. We report data for the remaining 96 participants. All participants are native speakers of American English. Participants were paid three dollars upon completion of the task.

### 9.2. Methods

Experiment VI is based on Experiments II and V. The morphological pattern is the plural, as in Experiment V. The extent of exposure is three times as great as in Experiment V: The plural pattern is trained with 18 (instead of 6) items and 4 conversation partners, and it is tested with these 18 items and 18 previously unseen items. There are no unseen conversational partners in the test phase, as in Experiment II. Since the focus is on the effect of familiarity with training items and since including new conversation partners as well would have prolonged the experiment to a large degree, we only included conversation partners seen in training in the test. We use the same conversation partners as in experiments I, II, and IV: the *woman* and the *man*. Our list of stimuli was expanded for Experiment VI (cf. Table 13). The main principle was to avoid adding syllables with consonant clusters which would upset the symmetry of the concatenated words. This was achieved by adding “ng,” the English consonant letter for the velar nasal, to the set of available syllable codas.

**Table 13:** **Stimuli set, Experiment VI**.

fos	ruk	wik	ril
fol	pil	fel	tos
fon	tang	rong	fok
tel	fek	rel	tas
feng	fong	ros	wis
wal	tal	rek	pek
pung	fus	tol	rik
wun	rak	ren	ral
tus	wus	rok	tok

Experiment VI uses the adult *woman* and *man* conversation partners in *front* and *side* view.

### 9.3. Hypotheses

Our hypotheses were based on the the results of Experiments II and V. We expected that participants would learn the contextual association more easily in the *gender* condition than in the *view* condition. We also expected that they would generalize to new items. In Experiment VI, we were also seeking a more in depth understanding of individual success rates. In addition to evaluating the effects of participant age and gender, we asked whether the lengthened training phase improves the success rate, compared to the previous experiments, and whether it affects the distribution of the good learners vs. poor learners.

### 9.4. Results

As in the previous experiment, we first analyzed the data from Experiment VI and then went on to compare it with Experiment II, which has a similar setup but shorter training.

Similar to most previous experiments, training takes longer with the non-salient cue (*view*) than with the salient cue (*gender*) (*W* = 584, *p* < 0.001).

**Figure 13:**
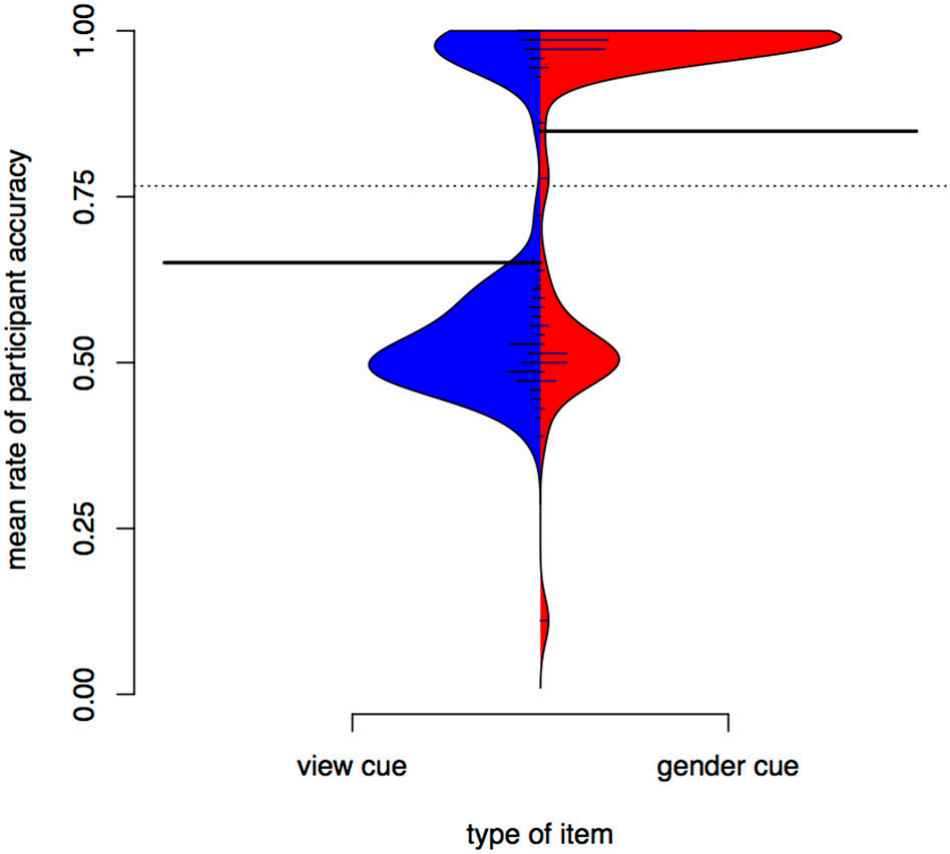
**Distributions of participant responses on test items in the ***view*** and ***gender*** conditions, Experiment VI**. Black horizontal bars show the mean accuracy for each condition. The dotted line shows the overall mean. Small horizontal lines show individual values; longer if multiple individuals have the same average.

The mean rate of participant accuracy in test can be seen in Figure 13.

We fit a regression model on the test phase of Experiment VI following the same procedure as in the previous experiments. The model summary can be seen in Table 14. The only significant predictor is cue type (β = 2.45, *se* = 0.51, *p* < 0.001).

**Table 14:** **Best model summary, Experiment VI**.

**Formula: correct** ~ **cue type** + **(1** | **participant)**				
	**Estimate**	**Std. Error**	***z*****-value**	**Pr(**>|**z**|**)**
(Intercept)	1.18	0.38	3.10	0.00
Cue type = gender	2.45	0.51	4.77	0.00

We then compare the test data from Experiment VI to test data from Experiment II. Experiment II constitutes the best comparison since it also has no new conversation partners in test. The pattern type is the diminutive rather than the plural, but Experiment V provided little evidence that this would be a relevant dimension.

We merge the two test datasets to see whether test accuracy in Experiment VI (which has 18 training items) is better than in Experiment II (which has 6 training items), and whether this has any interaction with cue type (*gender* or *view*).

We stepwise fit a binomial mixed-effects regression model on the test data using response as an outcome variable and item presence in training, cue type, and participant age and gender as predictors, with a participant random intercept. The summary of the best model can be seen in Table 15,^3^.

**Table 15:** **Best model summary, Experiments II and VI**.

**Formula: correct** ~ **training length** ^*^ **cue type** + **(1** | **participant)**				
	**Estimate**	**Std. Error**	***z*****-value**	**Pr(**>|**z**|**)**
(Intercept)	1.64	0.34	4.82	0.00
Training length = 18 items	1.94	0.46	4.24	0.00
Cue type = gender	−0.86	0.48	−1.78	0.08
Training length = 18 items:cue type = gender	−1.56	0.67	−2.34	0.02

**Figure 14:**
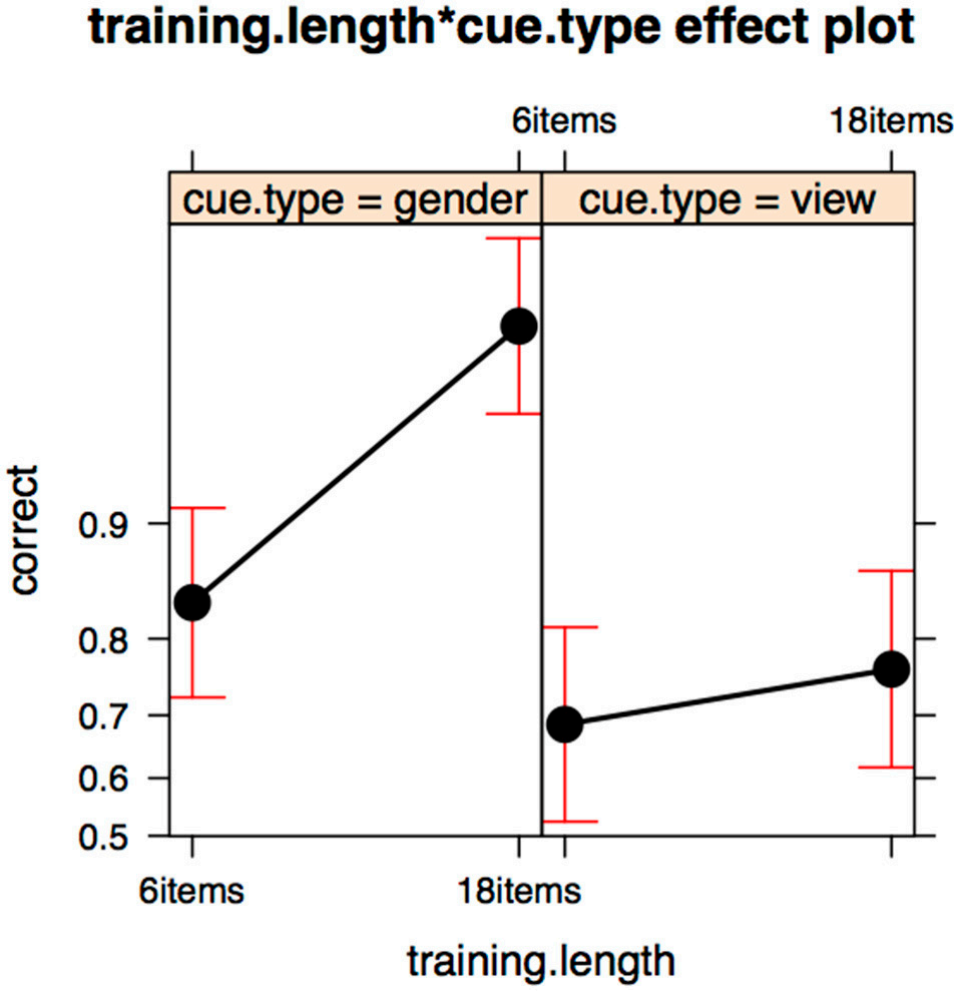
**Interaction term from the regression model: participant trained with the gender cue benefit from longer training in Experiment VI**.

We see that cue type has a strong positive effect on test accuracy. Training length matters. Participants who go through longer training are more accurate in the test phase. However, this effect is mostly carried by the *gender* cue: longer training is beneficial to those who are trained with the gender distinction. The effect plot can be seen in Figure 14.

If we tabulate good learners and poor learners across conditions and compare the results to Experiment II (which is similar in structure but has a shorter training phase), we find that the ratio of good learners increases with increased training (cf. Table 16—there are more good learners in Experiment VI than in Experiment II).

**Table 16:** **“Good” learners across cue type, Experiments II and VI**.

	**Experiment II**	**(6 training items)**	**Experiment VI**	**(18 training items)**
	**View cue**	**Gender cue**	**View cue**	**Gender cue**
Good learners	5	25	12	41
Poor learners	40	27	28	15

### 9.5. Discussion

Based on the results of experiments I–IV, we hypothesized that two types of information are available to participants in this experimental paradigm, the stem (the linguistic context) and the suffix (the non-linguistic context). If the non-linguistic context is salient, it is more readily available as a factor in variation. What remaines unclear is whether participants then decide to focus on the stem or the suffix (resulting in less or more success in the experiment) at random or based on specific learning strategies. Experiment VI shows that if we increase the training set, more participants are able to determine the relevant cue for the linguistic pattern (the non-linguistic context). This indicates that, if participants adopt a learning strategy (e.g., focussing on the prompt picture or on the stem), some of them are able to update it based on evidence that it does not yield good results. Increasing the amount of evidence available by lengthening the training phase enables more participants to modify their strategy and succeed at the task.

Age has no effect on test accuracy for participants with extended training length. This indicates that training can overcome the age effect. It is true, however, that the effect of age in our experiments is not robust with this sample size. This means that we have to be very cautious in interpreting the lack of an effect here. Ultimately, the relationship of training length and age could only be tested with a larger sample, which is outside the scope of this paper.

## 10. Summary

We have given a review of the literature to show that the non-linguistic context is extremely influential in learning linguistic constructions. Indeed, language use is shaped to a large degree by the social context. However, the link between contextual language learning and the observed structural complexity of social language use is far from completely understood.

We presented a series of artificial language learning experiments in which learning takes place in different contexts, which have different degrees of social-cognitive salience. The experiments were designed to investigate whether the relative social salience of contextual cues is relevant to learning a language pattern and whether this pattern is generalized to new lexical items and contexts. We hypothesized that participants would fare better at learning the link between the type of conversation partner and morphological pattern if the categorization of conversation partners was socially salient. We also assumed that this salient link would be generalized to new items and new conversation partners.

We found that participants learned the association of two morphological patterns (a diminutive suffix and a plural suffix) with conversation partner identity or gender, much as they learned the linguistic pattern that we used as a baseline (a cooccurrence constraint between the stem and suffix vowels).

Successful learners of the contextual association generalized well to new items. However, learning contextual associations was overall rather difficult for the participants. There were substantial individual differences in learning. As in the survey of statistical learning in various domains by Siegelman and Frost (2015), we found that people vary in their individual ability to learn from training data—some people have high accuracy, and others perform worse.

The test data distributions generally showed two distinct modes, one for “good learners” and one for “poor learners.” The adaptive tracking training enabled us to examine the differences between good learners and poor learners in more depth. Good learners finished the training phrase faster, suggesting that they identified and focused on the relevant cue better than poor learners did. However, even participants who were “good learners” needed to make a number of mistakes to learn the pattern. The distributions of training trial counts for “good learners” reveal that training took good learners longer than would be needed for a player who plays ideally. This means that each of them had to make at least a few mistakes before they learned the pattern. With the lengthened training phrase in Experiment VI, a greater number of opportunities to notice the relevant cue had the result that a greater number of participants responded to failure by readjusting their focus, ultimately patterning as good learners in the test phase.

An important result of these experiments is the relative success for different non-linguistic contextual dimensions. Social salience is very important. When the link between the conversation partner and the item appeared relatively accidental (side-facing, vs. front-facing), the association was very difficult to learn. When the link was socially coherent and interpretable (conversation partner gender), the learning task was considerably easier (Experiments III, V, and VI). Participants learn relatively easily, for example, that a particular adult female calls a small *fen* a *fenwun*, whereas a different person—a male—calls it a *fentas*. Participants orient early to the contextual cue of gender, and easily generalize this both to new items and to other conversation partners (Experiment III). This is true even when little evidence of generality is actually given. That is, exposure to just one female partner saying *fenwun* (in two views) leads to the hypothesis that all females would prefer *fenwun* to *fentas*.

Another aspect of learning is the competition between the linguistic context and non-linguistic context. In Experiment I, where participants need to focus on the linguistic context (the prompt name), familiar items (ones seen in training) are chosen more accurately in test. In Experiments II and III, where participants need to focus on the non-linguistic context (along with the suffix), this effect is absent. This remains true even for the “poor learner” participant group—those who did not seem to pin down the relevant contextual difference (conversation partner gender or spatial orientation).

In Experiment IV, we see that participants who focus on the suffix in test also finish training faster. This, in turn, supports the interpretation that the two types of information (stem and suffix) are competing with each other. Concentrating on the suffix is the key to success. Experiment V shows that this learning strategy is robust (applies for learning both the plural and the diminutive) while Experiment VI shows that the choice of stem or suffix as the main locus of attention is not fixed. With increased training, more participants figure out the relevant dimension and respond like “good learners” in the test phase.

These results can be compared with the results of Lleras and Von Mühlenen (2004). They find that, in a learning experiment, where contextual cues correlate with tasks, participants that employ an “active” searching strategy, and focus on the task itself, do not rely on contextual cues. The participants in our experiments follow an analogous pattern. Those who focus on the stem ignore social contextual cues. For the baseline Experiment I, the social contextual information is irrelevant and focusing on the stem would lead to success; but in the other experiments, focusing on the stem would cause the participant to overlook the information that is actually relevant to the task. The interesting point is that *whether* they focus on the stem or the suffix depends on the kind of social contextual cue present. They are more likely to rely on the social contextual cue if it is salient.

Taken together, the results of these experiments provide solid evidence that adults are able to learn contextual meaning, and that they orient more toward contextual information that is socially salient and relevant than to contextual variation that appears accidental.

## 11. General discussion

The focus of this article is learning associations between a morphological pattern and a non-linguistic context. The main question is how the social salience of the context influences success in learning.

We contrasted the learning of two cues, one of which is socially salient (*gender*) and one of which is not (*view*), showing that the former is learned more easily than the latter. As we note in Section 3, the perceptual differences between the images are unlikely to affect their categorization.

Of course, the forced-choice paradigm has its limitations. When we say that participants were better at learning the *gender* cue, this needs to be interpreted within the context of the task. We do not know whether they learned it well enough to produce it unprompted, for example, nor do we know whether, outside of a forced-choice task, they would have preferred some unknown other response. The positive side of a forced-choice paradigm is that our results are easy to interpret statistically. But the results do open up further questions about how the results would pattern if a free-response paradigm was used. As we only contrast gender and view, a further question is whether these two conditions may vary on unknown dimensions other than salience. Further research using other images and other contrasts is therefore still required.

Social salience has a top-down effect. Prior experience teaches us that some differences are more important than others, and we pay more attention to these in linguistic categorization. The way we see the world, then, has a strong influence on our language use, resulting in the complex structures of indexicality discussed by sociolinguists on the population level. This article provides evidence that this influence is present on an individual level. The social salience of the images is likely to rely on more than “gender”, but it remains the core manipulation that participants react to. The manipulation appears to provide a very reliable effect, despite the simplicity of the experimental paradigm.

Our results indicate that participants give more accurate answers when they recognize the relevant distinction (e.g., female/male in the *gender* condition). This is broadly analogous to explicit social stereotypes in terms of recognizing both the pattern and the context, as well as the connection between the two. At the same time, it can also be extended to cases in which either the context or the pattern is recognized and negotiated explicitly. The former is typical of most cases of social-indexical variation, in which we know our conversation partner's principal characteristics. The latter is typical of word patterns in particular, such as the choice between the formal and the informal second person conjugation in French and German, and the dialect-specific vocabularies of English, German, French, and many other national languages.

Experiment I, as well as the combined analysis of Experiments II, III, and V, showed that older participants were more successful at both morpho-phonological and socio-indexical learning.

The age effect may arise because prior experience, increasing throughout the lifespan, has a beneficial effect on learning tasks, as proposed by Ramscar et al. (2014). As older participants were better at learning all associations presented (including the un-natural *view* association), this cannot be viewed as an effect of increased experience with socially relevant distinctions. Rather, it would have to be interpreted as an effect of increased general experience with learning socio-indexical and linguistic associations. The age effect may also arise in some way from the specific nature of our task. For example, if participants select the wrong answer, they get feedback. This feedback could provide them with information that are orienting to the wrong cue. There is some evidence that older participants make better use of feedback, especially in situations in which they are initially uncertain (Metcalfe et al., 2015).

It is important to note that the age range of our participants is restricted, when considered in the context of the literature on ageing. Only one of our 498 participants (with age data available) is over 70. The considerable literature on cognitive decline in ageing across a range of psychological tasks compares younger and older adults (usually over 70), with an assumption that speakers in the middle (i.e., 40–60) fall somewhere in between (Lachman, 2004). While the middle-aged group tends to be less studied, there are at least some studies which show improvement from younger adults to middle age, before declining again in older adults. Tasks where such an effect has been reported include everyday problem-solving (Thornton et al., 2013) and social problem-solving (D'Zurilla et al., 1998).

It is interesting to note that we see no age effect for the task with extended training (Experiment VI). This may suggest that, whatever the root of the age effect, additional practice can neutralize the benefits of increased age in this task.

Our results indicate a major role for social salience in the acquisition of contextual meaning in morphology. In our task, the contextual information is associated with the morphological pattern in the cognitive representation, and influences recall and generalization. Whether or not participants are overtly aware of the association, it is sufficient to nudge them in the correct direction in a two-way forced choice test. The fact that the gender-dependent association is learned better than an accidental association, and that performance slightly improves with age, reveals the role of prior knowledge and expectation about what aspects of the context may potentially be relevant. Many aspects of language vary according to the gender of the conversation partner, and in the participants' prior sociolinguistic learning, the gender of the conversation partner will have been relevant many times. Foulkes (2010) hypothesizes that some types of indexical properties should be more readily transmitted than others, based on the frequency with which they have been relevant in individuals' past experience. He identifies gender as one of the earliest learned socio-indexical associations. Children as young as 6 months, for example, preferentially match sex-cued voices and faces (Walker-Andrews et al., 1991). It is likely this considerable prior experience that facilitates a ready generalization across conversation partners.

## 12. Conclusion

Our paradigm demonstrates differences in adult learning of socially salient vs. accidental non-linguistic contextual cues. It also reveals a number of questions about the way we learn contextual associations of higher level linguistic structures. Does a varying non-linguistic context aid the learning of a linguistic pattern? Do we learn the diminutive more easily, for example, if we are exposed to more types of conversation partners who use it? What is the effect of attention to particular linguistic patterns and non-linguistic contexts? Does variance in a non-linguistic difference that we explicitly attend to aid language learning? Finally, amongst socially salient non-linguistic cues, are some easier to learn than others? Is it easier to learn the association of a linguistic pattern with gender, for example, than with age? These questions remain to be answered by follow-up research.

Our controlled laboratory experiments are, of course, still many worlds apart from the type of complex socio-contextual learning and generalization that occurs in every day interaction and language acquisition. However they do provide some first steps toward shedding some light on the complex cognitive mechanisms that must be at play in such learning. Whether an associative pattern is attended to, learned and recreated by a speaker will be affected by a range of factors—including who that individual is, how socially salient the relevant context is, and how much the learner is exposed to that association. Our experiments have shown that individual variability in individual listeners, the salience of socio-contextual associations, and differing patterns of exposure, likely all play some role in affecting socio-contextual learning in morphology.

## Author contributions

PR designed and ran the experiments, performed statistical analysis, and wrote up the results. JH and JP contributed to the design, provided feedback on the experiments and analysis and contributed to writing up the manuscript.

## Funding

This project was made possible through the support of a grant to Northwestern University from the John Templeton Foundation (Award ID 36617). The opinions expressed in this publication are those of the author(s) and do not necessarily reflect the views of the John Templeton Foundation. JH was also supported by a Royal Society of New Zealand Rutherford Foundation Grant (Grant Number E5909).

### Conflict of interest statement

The authors declare that the research was conducted in the absence of any commercial or financial relationships that could be construed as a potential conflict of interest.

## Footnotes

^1^Due to the use of the simplified adaptive tracking paradigm, a player who guesses randomly on all training targets would need 518 trials, on the average, to finish training. The high number is due to the fact that, in the adaptive tracking paradigm, an incorrect answer returns the previous trial, and an incorrect answer to that throws the participant back even further.

^2^We initially ran 40 participants in this experiment. A reviewer pointed out that the lower participant count is problematic given that we compare this experiment to Experiment III. We have then run additional participants for Experiment V. Regression analysis shows no difference in the performance of the first and second batch in Experiment V.

^3^The way the experimental platform assigns participants to conditions has a slight random element, and one of the conditions has considerably fewer participants in it. In order to make sure that our results are robust, we re-fit our model on a subset of the test data with 39 participants in each condition. The main effects did not change considerably.

## References

[B1] Altmann E. G. Pierrehumbert J. B. Motter A. E. (2011). Niche as a determinant of word fate in online groups. PLoS ONE 6:e19009. 10.1371/journal.pone.0019009 21589910 PMC3093376

[B2] Baayen R. H. Hendrix P. Ramscar M. (2013). Sidestepping the combinatorial explosion: an explanation of *n*-gram frequency effects based on naive discriminative learning. Lang. Speech. 56, 329–347. 10.1177/0023830913484896 24416960

[B3] Bates D. Maechler M. Bolker B. (2012). lme4: Linear Mixed-Effects Models using S4 Classes. R Package.

[B4] Bauer L. (1997). Evaluative morphology: in search of universals. Stud. Lang. 21, 533–575. 10.1075/sl.21.3.04bau

[B5] Baxter G. J. Blythe R. A. Croft W. McKane A. J. (2009). Modeling language change: an evaluation of Trudgill's theory of the emergence of New Zealand English. Lang. Variat. Change 21, 257–296. 10.1017/S095439450999010X

[B6] Becker M. Ketrez N. Nevins A. (2011). The surfeit of the stimulus: analytic biases filter lexical statistics in Turkish laryngeal alternations. Language 87, 84–125. 10.1353/lan.2011.0016

[B7] Beckner C. Rácz P. Brandstetter J. Hay J. Bartneck C. (2016). Participants conform to humans but not to humanoid robots in an English past tense formation task. J. Lang. Soc. Psychol. 35, 158–179. 10.1177/0261927X15584682

[B8] Brooks P. J. Kwoka N. Kempe V. (2016). Distributional effects and individual differences in L2 morphology learning. Lang. Learn. [Epub ahead of print]. 10.1111/lang.12204

[B9] Campbell-Kibler K. (2011). Intersecting variables and perceived sexual orientation in men. Amer. Speech 86, 52–68. 10.1215/00031283-1277510

[B10] Cheshire J. (2002). Sex and gender in variationist research, in Handbook of Language Variation and Change, eds Chambers J. K. Trudgill P. Schilling N. (Hoboken NJ: Wiley Blackwell), 423–443.

[B11] Chun M. M. Jiang Y. (1998). Contextual cueing: implicit learning and memory of visual context guides spatial attention. Cogn. Psychol. 36, 28–71. 10.1006/cogp.1998.0681 9679076

[B12] Coupland J. Coupland N. Giles H. (eds.). (1991). Accommodation theory. communication, context and consequences, in Contexts of Accommodation (Cambridge; Paris: Cambridge University Press, Éditions de la Maison des Sciences de l–homme), 1–68.

[B13] Crump M. J. McDonnell J. V. Gureckis T. M. (2013). Evaluating Amazon's Mechanical Turk as a tool for experimental behavioral research. PLoS ONE 8:e57410. 10.1371/journal.pone.0057410 23516406 PMC3596391

[B14] Denton S. E. Kruschke J. K. Erickson M. A. (2008). Rule-based extrapolation: a continuing challenge for exemplar models. Psychon. Bull. Rev. 15, 780–786. 10.3758/PBR.15.4.780 18792504

[B15] Dixon R. M. W. (1980). The Languages of Australia. Cambridge: Cambridge University Press.

[B16] Docherty G. J. Langstrof C. Foulkes P. (2013). Listener evaluation of sociophonetic variability: probing constraints and capabilities. Linguistics 51, 355–380. 10.1515/ling-2013-0014

[B17] Drager K. (2010). Sociophonetic variation in speech perception. Lang. Linguist. Compass 4, 473–480. 10.1111/j.1749-818X.2010.00210.x

[B18] D'Zurilla T. J. Maydeu-Olivares A. Kant G. L. (1998). Age and gender differences in social problem-solving ability. Pers. indiv. Diff. 25, 241–252.

[B19] Eckert P. (2000). Linguistic Variation as Social Practice: The Linguistic Construction of Identity in Belten High. Hoboken, NJ: Wiley-Blackwell.

[B20] Eckert P. (2008). Variation and the indexical field. J. Sociolinguist. 12, 453–476. 10.1111/j.1467-9841.2008.00374.x

[B21] Eckert P. (2012). Three waves of variation study: the emergence of meaning in the study of sociolinguistic variation. Ann. Rev. Anthropol. 41, 87–100. 10.1146/annurev-anthro-092611-145828

[B22] Fagyal Z. Swarup S. Escobar A. M. Gasser L. Lakkaraju K. (2010). Centers and peripheries: network roles in language change. Lingua 120, 2061–2079. 10.1016/j.lingua.2010.02.001

[B23] Fedzechkina M. Jaeger T. F. Newport E. L. (2012). Language learners restructure their input to facilitate efficient communication. Proc. Natl. Acad. Sci. U.S.A. 109, 17897–17902. 10.1016/j.lingua.2010.02.001 23071337 PMC3497763

[B24] Foulkes P. (2010). Exploring social-indexical knowledge: a long past but a short history. Lab. Phonol. 1, 5–39. 10.1515/labphon.2010.003

[B25] Foulkes P. Docherty G. (2006). The social life of phonetics and phonology. J. Phonet. 34, 409–438. 10.1016/j.wocn.2005.08.002

[B26] Foulkes P. Hay J. B. (2015). The emergence of sociophonetic structure, in The Handbook of Language Emergence, eds MacWhinney B. O'Grady W. (Hoboken, NJ: Wiley Blackwell), 292.

[B27] Gelman A. Hill J. (2006). Data Analysis Using Regression and Multilevel/Hierarchical Models. Cambridge: Cambridge University Press.

[B28] German J. S. Carlson K. Pierrehumbert J. B. (2013). Reassignment of consonant allophones in rapid dialect acquisition. J. Phonet. 41, 228–248. 10.1016/j.wocn.2013.03.001

[B29] Gluck M. A. Myers C. E. (2001). Gateway to Memory: An Introduction to Neural Network Modeling of the Hippocampus and Learning. Cambridge, MA: MIT Press.

[B30] Godden D. R. Baddeley A. D. (1975). Context-dependent memory in two natural environments: on land and underwater. Br. J. Psychol. 66, 325–331. 10.1111/j.2044-8295.1975.tb01468.x

[B31] Gómez R. L. (2002). Variability and detection of invariant structure. Psychol. Sci. 13, 431–436. 10.1111/1467-9280.00476 12219809

[B32] Goujon A. Didierjean A. Thorpe S. (2015). Investigating implicit statistical learning mechanisms through contextual cueing. Trends Cogn. Sci. 19, 524–533. 10.1016/j.tics.2015.07.009 26255970

[B33] Grossberg S. (1987). Competitive learning: from interactive activation to adaptive resonance. Cogn. Sci. 11, 23–63. 10.1111/j.1551-6708.1987.tb00862.x

[B34] Hay J. Walker A. (2013). Skewed experience with words affects lexical access patterns, in Variation and Language Processing (VALP) 2 (Christchurch).

[B35] Hay J. B. Drager K. (2007). Sociophonetics. Ann. Rev. Anthropol. 36, 89–103. 10.1146/annurev.anthro.34.081804.120633

[B36] Hay J. B. Drager K. (2010). Stuffed toys and speech perception. Linguistics 48, 865–892. 10.1515/ling.2010.027

[B37] Henderson L. Devine K. W. Gaskell M. (2015). When the daffodat flew to the intergalactic zoo: off-line consolidation is critical for word learning from stories. Dev. Psychol. 51, 406–417. 10.1037/a0038786 25642602

[B38] Horton W. S. Gerrig R. J. (2002). Speakers' experiences and audience design: knowing when and knowing how to adjust utterances to addressees. J. Mem. Lang. 47, 589–606. 10.1016/S0749-596X(02)00019-0

[B39] Horton W. S. Gerrig R. J. (2005). The impact of memory demands on audience design during language production. Cognition 96, 127–142. 10.1016/j.cognition.2004.07.001 15925573

[B40] Itti L. Koch C. Niebur E. (1998). A model of saliency-based visual attention for rapid scene analysis. IEEE Trans. Patt. Anal. Mach. Intell. 20, 1254–1259. 10.1109/34.730558

[B41] Johnson K. Strand E. A. D'Imperio M. (1999). Auditory–visual integration of talker gender in vowel perception. J. Phonet. 27, 359–384.

[B42] Jurafsky D. (1993/2012). Universals in the semantics of the diminutive, in Annual Meeting of the Berkeley Linguistics Society, Vol. 19 (Berkeley, CA). 10.3765/bls.v19i1.1531

[B43] Kraljic T. Brennan S. E. Samuel A. G. (2008a). Accommodating variation: dialects, idiolects, and speech processing. Cognition 107, 54–81. 10.1016/j.cognition.2007.07.013 17803986 PMC2375975

[B44] Kraljic T. Samuel A. G. (2006). Generalization in perceptual learning for speech. Psychon. Bull. Rev. 13, 262–268. 10.3758/BF03193841 16892992

[B45] Kraljic T. Samuel A. G. Brennan S. E. (2008b). First impressions and last resorts. How listeners adjust to speaker variability. Psychol. Sci. 19, 332–338. 10.1111/j.1467-9280.2008.02090.x 18399885

[B46] Kruisinga E. (1942). Diminutieve en Affektieve Suffixen in de Germaanse Talen. Groningen: Noord-Hollandsche Uitgevers Maatschappij.

[B47] Labov W. (2001). Principles of Linguistic Change: Social Factors. Hoboken, NJ: Wiley-Blackwell.

[B48] Lachman M. E. (2004). Development in midlife. Annu. Rev. Psychol. 55, 305–331. 10.1146/annurev.psych.55.090902.141521 14744218

[B49] Langstrof C. (2014). Sociophonetic Learning in L1 and L2. Freiburg: Habilitation, University of Freiburg.

[B50] Leek M. R. (2001). Adaptive procedures in psychophysical research. Percept. Psychophys. 63, 1279–1292. 10.3758/BF03194543 11800457

[B51] Leung J. H. Williams J. N. (2012). Constraints on implicit learning of grammatical form-meaning connections. Lang. Learn. 62, 634–662. 10.1111/j.1467-9922.2011.00637.x

[B52] Leys C. Ley C. Klein O. Bernard P. Licata L. (2013). Detecting outliers: do not use standard deviation around the mean, use absolute deviation around the median. J. Exp. Soc. Psychol. 49, 764–766. 10.1016/j.jesp.2013.03.013

[B53] Lleras A. Von Mühlenen A. (2004). Spatial context and top-down strategies in visual search. Spat. Vis. 17, 465–482. 10.1163/1568568041920113 15559114

[B54] Mathworks (2016). MATLAB Version 9.1.0.441655 (R2016b). Natick, MA: The Mathworks, Inc.

[B55] Maye J. Aslin R. N. Tanenhaus M. K. (2008). The weckud wetch of the wast: lexical adaptation to a novel accent. Cogn. Sci. 32, 543–562. 10.1080/03640210802035357 21635345

[B56] Metcalfe J. Casal-Roscum L. Radin A. Friedman D. (2015). On teaching old dogs new tricks. Psychol. Sci. 26, 1833–1842. 10.1177/0956797615597912 26494598 PMC4679660

[B57] Milroy J. Milroy L. (1993). Mechanisms of change in urban dialects: the role of class, social network and gender. Int. J. Appl. Linguist. 3, 57–77. 10.1111/j.1473-4192.1993.tb00043.x

[B58] Milroy L. (1980). Language and Social Networks. Oxford: Oxford University Press.

[B59] Munro R. Bethard S. Kuperman V. Lai V. T. Melnick R. Potts C. (2010). Crowdsourcing and language studies: the new generation of linguistic data, in Proceedings of the NAACL HLT 2010 Workshop on Creating Speech and Language Data with Amazon's Mechanical Turk (Association for Computational Linguistics), 122–130.

[B60] Needle J. N. Pierrehumbert J. B. Hay J. B. (2015). Effects of pseudomorphology on the wordlikeness of pseudowords, in Architectures and Mechanisms for Language Processing.' 3 - 5 September. University of Malta Valletta (Los Angeles, CA).

[B61] Nosofsky R. M. (1988). Exemplar-based accounts of relations between classification, recognition, and typicality. J. Exp. Psychol. Learn. Mem. Cogn. 14:700 10.1037/0278-7393.14.4.700

[B62] Pele O. Werman M. (2010). The quadratic-chi histogram distance family, in European Conference on Computer Vision (New York, NY: Springer), 749–762.

[B63] Pierrehumbert J. B. (2006). The next toolkit. J. Phonet. 34, 516–530. 10.1016/j.wocn.2006.06.003

[B64] Pierrehumbert J. B. Bent T. Munson B. Bradlow A. R. Bailey J. M. (2004). The influence of sexual orientation on vowel production. J. Acoust. Soc. Am. 116, 1905–1908. 10.1121/1.1788729 15532622

[B65] Pierrehumbert J. B. Stonedahl F. Daland R. (2014). A model of grassroots changes in linguistic systems. arXiv preprint arXiv:1408.1985.

[B66] Preston D. R. (1996). Whaddayaknow?: The modes of folk linguistic awareness. Lang. Awareness 5, 40–74. 10.1080/09658416.1996.9959890

[B67] Qian T. Jaeger T. Aslin R. N. (2014). Implicit Learning in a Non-stationary Environment: Knowing When It's Groundhog Day. MS, University of Rochester (Accessed March 17, 2014).

[B68] R Core Team (2016). R: A Language and Environment for Statistical Computing. Vienna: R Foundation for Statistical Computing.

[B69] Rácz P. (2013). Salience in Sociolinguistics: A Quantitative Approach. Berlin: De Gruyter - Mouton.

[B70] Ramscar M. Hendrix P. Shaoul C. Milin P. Baayen H. (2014). The myth of cognitive decline: non-linear dynamics of lifelong learning. Top. Cogn. Sci. 6, 5–42. 10.1111/tops.12078 24421073

[B71] Ratcliff R. (1978). A theory of memory retrieval. Psychol. Rev. 85:59 10.1037/0033-295X.85.2.59 3406246

[B72] Roberts G. (2008). Language and the free-rider problem: an experimental paradigm. Biol. Theory 2, 174–183. 10.1162/biot.2008.3.2.174

[B73] Rost G. C. McMurray B. (2009). Speaker variability augments phonological processing in early word learning. Dev. Sci. 12, 339–349. 10.1111/j.1467-7687.2008.00786.x 19143806 PMC3011987

[B74] Säily T. Suomela J. (2009). Comparing type counts: the case of women, men and *-ity* in early English letters, in Corpus Linguistics: Refinements and Reassessments, eds Renouf A. Kehoe A. (Amsterdam: Rodopi), 87–109.

[B75] Schumacher R. A. Pierrehumbert J. B. LaShell P. (2014). Reconciling inconsistency in encoded morphological distinctions in an artificial language, in Proceedings of the 36th Annual Conference of the Cognitive Science Society. Cognitive Science Society (Quebec City, QC).

[B76] Siegelman N. Frost R. (2015). Statistical learning as an individual ability: theoretical perspectives and empirical evidence. J. Mem. Lang. 81, 105–120. 10.1016/j.jml.2015.02.001 25821343 PMC4371530

[B77] Silverstein M. (2009). The limits of awareness, in Linguistic Anthropology: A Reader, Vol. 1, ed Duranti A. (Oxford: Blackwell), 382–401.

[B78] Smith S. M. Vela E. (2001). Environmental context-dependent memory: a review and meta-analysis. Psychon. Bull. Rev. 8, 203–220. 10.3758/BF03196157 11495110

[B79] Soliz J. Giles H. (2014). Relational and identity processes in communication: a contextual and meta-analytical review of communication accommodation theory. Commun. Yearbook 38, 106–143. 10.1080/23808985.2014.11679160

[B80] Tagliamonte S. A. Roeder R. V. (2009). Variation in the English definite article: socio-historical linguistics in t'speech community. J. Sociolinguist. 13, 435–471. 10.1111/j.1467-9841.2009.00418.x

[B81] Thornton W. L. Paterson T. S. Yeung S. E. (2013). Age differences in everyday problem solving: the role of problem context. Int. J. Behav. Dev. 37, 13–20. 10.1177/0165025412454028

[B82] Van der Zande P. Jesse A. Cutler A. (2014). Cross-speaker generalisation in two phoneme-level perceptual adaptation processes. J. Phonet. 43, 38–46. 10.1016/j.wocn.2014.01.003

[B83] Von Ahn L. (2006). Games with a purpose. Computer 39, 92–94. 10.1109/MC.2006.196

[B84] Walker A. Hay J. B. (2011). Congruence between ‘word age’ and ‘voice age’ facilitates lexical access. Lab. Phonol. 2, 219–237. 10.1515/labphon.2011.007

[B85] Walker-Andrews A. S. Bahrick L. E. Raglioni S. S. Diaz I. (1991). Infants' bimodal perception of gender. Ecol. Psychol. 3, 55–75. 10.1207/s15326969eco0302_1

[B86] Wells J. C. (1982). Accents of English, Vol. 1 Cambridge: Cambridge University Press.

[B87] Wickham H. (2009). ggplot2: Elegant Graphics for Data Analysis. New York, NY: Springer-Verlag.

[B88] Yu C. Smith L. B. (2007). Rapid word learning under uncertainty via cross-situational statistics. Psychol. Sci. 18, 414–420. 10.1111/j.1467-9280.2007.01915.x 17576281

